# Use of an exotic host plant shifts immunity, chemical defense, and viral burden in wild populations of a specialist insect herbivore

**DOI:** 10.1002/ece3.8723

**Published:** 2022-03-16

**Authors:** Nadya D. Muchoney, M. Deane Bowers, Adrian L. Carper, Peri A. Mason, Mike B. Teglas, Angela M. Smilanich

**Affiliations:** ^1^ Program in Ecology, Evolution, and Conservation Biology University of Nevada Reno Nevada USA; ^2^ Department of Biology University of Nevada Reno Nevada USA; ^3^ Department of Ecology and Evolutionary Biology & Museum of Natural History University of Colorado Boulder Colorado USA; ^4^ Department of Agriculture, Veterinary and Rangeland Sciences University of Nevada Reno Nevada USA

**Keywords:** *Euphydryas phaeton*, immune response, iridoid glycosides, Junonia coenia densovirus, plant secondary metabolites, tritrophic interactions

## Abstract

Defense against natural enemies constitutes an important driver of herbivore host range evolution in the wild. Populations of the Baltimore checkerspot butterfly, *Euphydryas phaeton* (Nymphalidae), have recently incorporated an exotic plant, *Plantago lanceolata* (Plantaginaceae), into their dietary range. To understand the tritrophic consequences of utilizing this exotic host plant, we examined immune performance, chemical defense, and interactions with a natural entomopathogen (Junonia coenia densovirus, *Parvoviridae*) across wild populations of this specialist herbivore. We measured three immune parameters, sequestration of defensive iridoid glycosides (IGs), and viral infection load in field‐collected caterpillars using either *P*. *lanceolata* or a native plant, *Chelone glabra* (Plantaginaceae). We found that larvae using the exotic plant exhibited reduced immunocompetence, compositional differences in IG sequestration, and higher in situ viral burdens compared to those using the native plant. On both host plants, high IG sequestration was associated with reduced hemocyte concentration in the larval hemolymph, providing the first evidence of incompatibility between sequestered chemical defenses and the immune response (i.e., the “vulnerable host” hypothesis) from a field‐based study. However, despite this negative relationship between IG sequestration and cellular immunity, caterpillars with greater sequestration harbored lower viral loads. While survival of virus‐infected individuals decreased with increasing viral burden, it ultimately did not differ between the exotic and native plants. These results provide evidence that: (1) phytochemical sequestration may contribute to defense against pathogens even when immunity is compromised and (2) herbivore persistence on exotic plant species may be facilitated by sequestration and its role in defense against natural enemies.

## Graphical abstract

In this study, we examined the tritrophic consequences of exotic host plant use by a native insect herbivore by measuring immune performance, chemical defense, and interactions with a natural entomopathogen across wild populations. We found that herbivores using an exotic host plant exhibited reduced immunocompetence, differential sequestration of defensive phytochemicals, and higher in situ viral burdens compared to those using a native plant. These results illustrate that host range expansion can give rise to a multifaceted shift in herbivore defenses against natural enemies and provide insight into the interacting roles of immune defense and phytochemical sequestration in mediating tritrophic interactions in the wild.

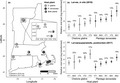

## 1 INTRODUCTION

The introduction of exotic species into ecosystems has profound impacts on the ecology and evolution of native organisms (Bezemer et al., 2014; Singer et al., 1993; Strauss et al., 2006; Sunny et al., 2015). In particular, the arrival of exotic plant species may create opportunities for the evolution of herbivore host range if these plants are incorporated into the diets of native herbivores (Bowers et al., 1992; Carroll & Fox, 2007; Forister et al., 2009; Singer et al., 1993). Incorporation of exotic host plant species is a common phenomenon in insect herbivores; for example, Graves and Shapiro (2003) reported that 34% of butterfly species in California utilize introduced plant taxa for either larval feeding or oviposition. Paradoxically, use of exotic host plants is overwhelmingly associated with negative effects on herbivore performance relative to native host plants in Lepidoptera (Yoon & Read, 2016), as indicated by metrics including development rate, weight, feeding efficiency, and survival (e.g., Bowers et al., 1992; Forister et al., 2009; Fortuna et al., 2013; Keeler & Chew, 2008). Therefore, the incorporation of exotic host plants into the diets of native herbivores frequently presents the question of which ecological benefits, if any, facilitate herbivore persistence on these species.

While optimization of herbivore performance, as measured by bitrophic indices focusing solely on the herbivore–plant interaction, often fails to explain patterns of host plant use (Forister et al., 2009; Mason et al., 2011), persistence on novel host plants involves an array of ecological, behavioral, and physiological factors beyond suitability for development (Forister & Wilson, 2013; Mason, 2016). In particular, herbivore interactions with natural enemies, including predators (Grosman et al., 2017; Murphy, 2004), parasitoids (Fortuna et al., 2013; Harvey & Fortuna, 2012), and pathogens (Cory & Hoover, 2006; Shikano, 2017) can differ substantially between native and exotic host plant species. As these interactions comprise a major source of mortality for insect herbivores (Hawkins et al., 1997), exploitation of enemy‐free or enemy‐reduced space may constitute an important driver of host range evolution (Bernays & Graham, 1988; Jeffries & Lawton, 1984; Singer & Stireman, 2005). Consideration of herbivore performance within a tritrophic framework, including attack by and defense against natural enemies, may therefore be essential for understanding the ecological mediators of persistence on exotic host plants (Fortuna et al., 2013; Harvey et al., 2010; Singer & Stireman, 2005).

In recent years, eco‐immunological research has highlighted the immune response as a critical physiological link between herbivore diet and interactions with natural enemies. Such studies have revealed substantial diet‐mediated variation in herbivore immune function (Singer et al., 2014), with repercussions for resistance against diverse natural enemies (reviewed in Smilanich & Muchoney, 2022). For example, several studies have documented positive effects of dietary protein content on caterpillar immune function, which have been linked to increased survival following bacterial or viral challenge in experimental settings (e.g., Cotter et al., 2019; Lee et al., 2006). In the wild, immunological variation may be driven by differences in plant quality (Diamond & Kingsolver, 2011; Klemola et al., 2008), macronutrient composition (Cotter et al., 2019; Lee et al., 2006), and/or secondary chemistry (Bukovinszky et al., 2009; Haviola et al., 2007; Smilanich, Dyer, Chambers, et al., 2009; Trowbridge et al., 2016). As the immune response provides insects with effective defenses against parasitoids (Carton et al., 2008; Smilanich, Dyer, & Gentry, 2009) and pathogens (Rantala & Roff, 2007; Washburn et al., 1996), host plant mediated variation in herbivore immunity may contribute to the ecological costs, or benefits, of host range expansion.

A second physiological link between herbivore diet and interactions with enemies is phytochemical sequestration. Some herbivores possess the ability to sequester toxic plant compounds and employ them in defense against natural enemies (Bowers, 1993; Nishida, 2002; Opitz & Müller, 2009). Use of exotic host plants, which may differ from native host plants in both composition and concentrations of secondary metabolites, can impact chemical defense in sequestering species, with ramifications for predator deterrence (Bowers, 1980; Knerl & Bowers, 2013) and fitness of internal parasites (Barbosa et al., 1991; De Roode et al., 2008; Ode, 2006; Singer et al., 2009). A less‐explored question is whether sequestration also indirectly affects herbivore–natural enemy interactions through modulation of the immune response. Diets containing high concentrations of secondary metabolites can suppress lepidopteran immune responses (Lampert & Bowers, 2015; Richards et al., 2012; Smilanich, Dyer, Chambers, et al., 2009), putatively rendering caterpillars more vulnerable to pathogens and parasitoids (but see Barthel et al., 2016; Garvey et al., 2021; Laurentz et al., 2012 for examples of positive immunological effects of secondary metabolites). This “vulnerable host” hypothesis (Smilanich, Dyer, Chambers, et al., 2009) has rarely been investigated, and typically only in laboratory settings, but offers potential for insight into the role of host plants in mediating physiological defenses against different types of natural enemies. In particular, characterizing the impacts of sequestration on herbivore immunity may reveal tradeoffs between investment in chemical and immunological forms of defense.

In this study, we examined the consequences of host range expansion by a native insect herbivore onto an exotic plant by measuring a suite of defenses employed by herbivores against natural enemies. We asked: can host plant mediated effects on insect defense help explain the paradox of herbivore persistence on exotic plants that confer relatively poor bitrophic performance? To address this question, we focused on a specialist lepidopteran herbivore, the Baltimore checkerspot (*Euphydryas phaeton* Drury, Nymphalidae), and an entomopathogen that occurs naturally in *E*. *phaeton* populations, Junonia coenia densovirus (*Parvoviridae*). *Euphydryas phaeton* recently incorporated the non‐native *Plantago lanceolata* L. into its host range (Stamp, 1979) and exhibits reduced performance on this exotic plant, compared to its primary native host plant, *Chelone glabra* L. (Bowers et al., 1992). Importantly, these two plant species differ in their composition of iridoid glycosides (Bowers et al., 1992; Duff et al., 1965), which are secondary metabolites that are sequestered by *E*. *phaeton* caterpillars (Bowers & Puttick, 1986) and have been shown to negatively impact the immune response of another specialist nymphalid butterfly species (Richards et al., 2012; Smilanich, Dyer, Chambers, et al., 2009). We combined approaches from the fields of eco‐immunology and chemical ecology to characterize the multifaceted effects of host plant use on herbivore defenses, focusing on the interacting roles of the immune response and phytochemical sequestration. We specifically addressed three questions: (1) Does use of an exotic host plant impact herbivore immunocompetence and/or sequestration? (2) Are higher levels of sequestration associated with reduced immunocompetence? (3) Do host plant mediated effects on immunocompetence and/or sequestration affect interactions with a natural pathogen? By evaluating variation in physiological defenses and viral infection across naturally occurring, wild herbivore populations, we provide insight into the tritrophic outcomes of host range expansion.

## 2 METHODS

### 2.1 Caterpillars, host plants, and virus

*Euphydryas phaeton* (Nymphalidae), the Baltimore checkerspot butterfly, is a univoltine North American herbivore that specializes on host plants containing iridoid glycosides (Bowers, 1980). Iridoid glycosides (hereafter, IGs) are monoterpenoid plant secondary metabolites that can be toxic and/or deterrent to generalist or non‐adapted specialist herbivores (Bowers & Puttick, 1988). *Euphydryas phaeton* caterpillars sequester IGs from host plants and retain them through the adult stage (Bowers & Puttick, 1986), rendering both larvae and butterflies unpalatable to predators (Bowers, 1980). Sequestration of IGs has been found to suppress larval immune responses in two specialist lepidopteran species (Lampert & Bowers, 2015; Richards et al., 2012; Smilanich, Dyer, Chambers, et al., 2009; but see Laurentz et al., 2012); however, the effects of IG sequestration on herbivore interactions with entomopathogens remain unknown.

The primary host plant for *E*. *phaeton* in the northeastern US is *Chelone glabra* (Plantaginaceae), white turtlehead, a native, long‐lived, perennial herb (Pennell, 1935). *Chelone glabra* primarily contains the IG catalpol, and may also contain smaller amounts of aucubin (Bowers et al., 1992), which is the chemical precursor of catalpol (Damtoft et al., 1983). Over the past 40 years, researchers have documented an expansion of *E*. *phaeton's* host range to include a non‐native plant, *Plantago lanceolata* (Plantaginaceae), narrowleaf plantain (Bowers et al., 1992; Stamp, 1979). This short‐lived, perennial herb was introduced to North America during the 19th century (Cavers et al., 1980). *Plantago lanceolata* also contains IGs (Bowers & Stamp, 1992), consisting of mainly aucubin with smaller amounts of catalpol (Duff et al., 1965; Fajer, 1989). Where these two host plant species co‐occur, *E*. *phaeton* may utilize both plants (Bowers & Richardson, 2013), while some populations use *P*. *lanceolata* or *C*. *glabra* exclusively (Bowers et al., 1992; Stamp, 1979). Despite this host range expansion, *E*. *phaeton* prefers the native *C*. *glabra* over *P*. *lanceolata* for both larval feeding and oviposition (Bowers et al., 1992). This is likely driven in part by costs associated with the exotic host plant, including reduced performance (Bowers et al., 1992) and increased palatability to predators (Bowers, 1980). However, *E*. *phaeton* population growth rates can be higher on *P*. *lanceolata* (Brown et al., 2017), suggesting that use of this exotic plant may entail both costs and benefits.

Junonia coenia densovirus (hereafter, JcDV) is a non‐enveloped, single‐stranded DNA virus in the family *Parvoviridae* (*Densovirinae*: *Lepidopteran protoambidensovirus 1*). Though first identified in *Junonia coenia* (Nymphalidae), JcDV is capable of infecting Lepidoptera in the Bombycidae, Erebidae, Noctuidae, and Nymphalidae in a laboratory setting (Mutuel et al., 2010; Resnik & Smilanich, 2020; Rivers & Longworth, 1968). Larvae become infected by JcDV through ingestion of contaminated food, after which viral particles cross the midgut and replicate in tracheae, hemocytes, visceral muscle, and epidermis (Mutuel et al., 2010; Wang et al., 2013). Infection may result in hypoxia, molting and metamorphosis failure, and death; however, pathogenesis is dose‐dependent and does not always result in mortality (Mutuel et al., 2010; Smilanich et al., 2018). Despite its potentially broad host range, little is known of JcDV in the wild; this study represents the first record of JcDV infection in the focal herbivore species, *E*. *phaeton*, and the first investigation of its occurrence in wild populations of any host species.

### 2.2 Experiment overview

To compare caterpillar immune responses, IG sequestration, and JcDV prevalence and infection loads across populations using the native and exotic host plant, we sampled caterpillars from wild *E*. *phaeton* populations in May 2016 and 2017. Caterpillars were brought to the University of Nevada, Reno, where they underwent a series of immune assays. In 2016, larvae were freeze‐killed following immune assessment to evaluate in situ relationships between host plant use, immune performance, IG sequestration, and viral infection. In 2017, caterpillars were *not* freeze‐killed following immune assessment, but reared out to ascertain the effects of host plant use, larval immune responses, and viral infection on survival. In both years, postmortem insects were screened for JcDV, and viral loads of infected individuals were quantified.

### 2.3 Population sampling

**FIGURE 1:**
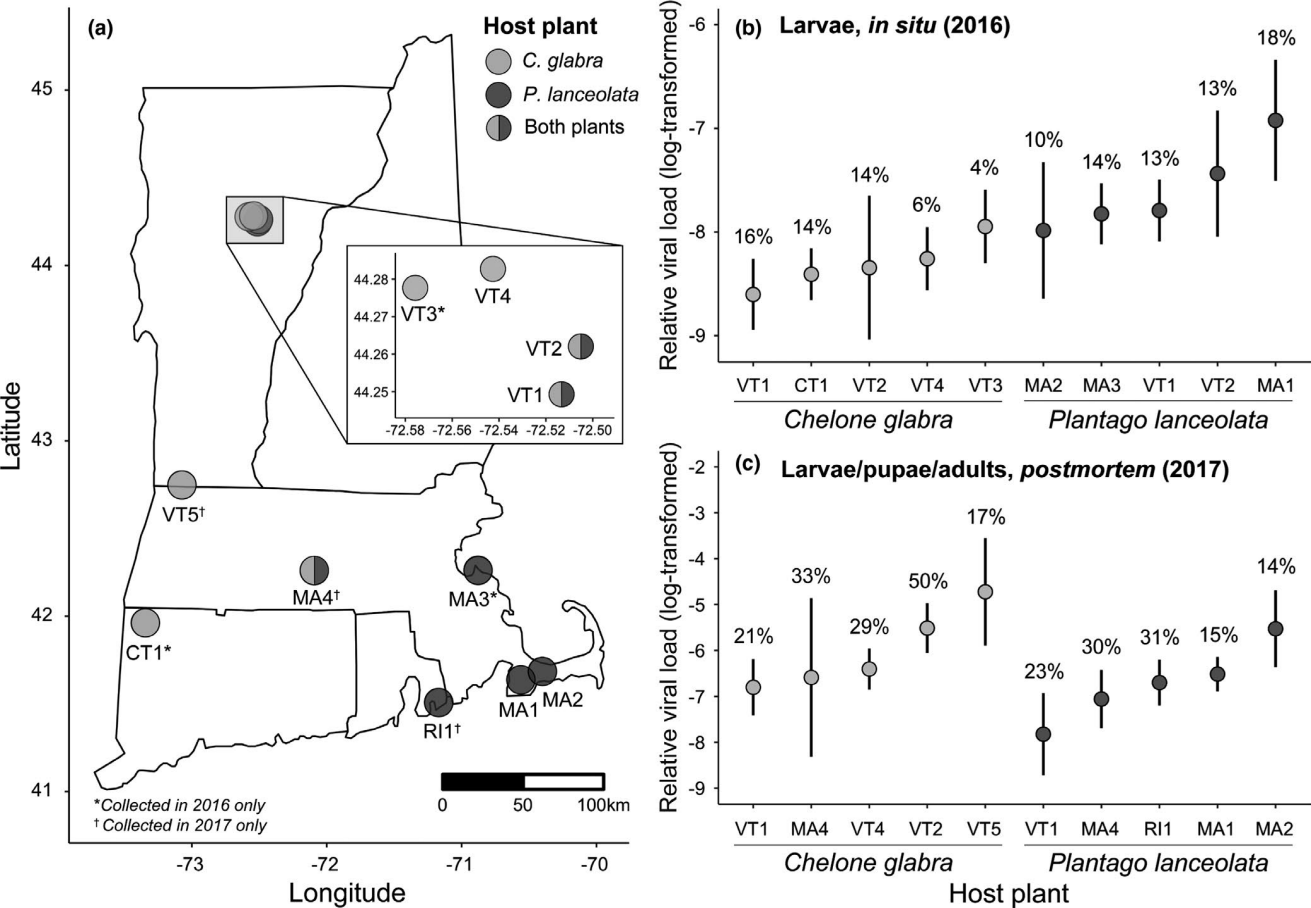
(a) Locations of sites in Connecticut (CT1), Massachusetts (MA1–4), Rhode Island (RI1), and Vermont (VT1–5) from which *Euphydryas phaeton* caterpillars were sampled. At each site, either the native host plant (*Chelone glabra*), exotic host plant (*Plantago lanceolata*), or both host plants were utilized. Caterpillars were collected in 2016 and 2017 unless otherwise indicated. (b, c) Junonia coenia densovirus loads of freeze‐killed larvae in 2016 (b) and lab‐deceased larvae, pupae, and adults in 2017 (c) across sites. Points represent mean viral load ± SE, with site‐level JcDV frequency (% infected individuals) above each point. Based on linear mixed‐effects models with an alpha level of 0.05, in situ JcDV loads were significantly higher in larvae using *P*. *lanceolata* (b), but postmortem JcDV loads did not differ significantly based on host plant (c)

*Euphydryas phaeton* caterpillars were collected from populations located throughout the northeastern US (Figure 1a). In 2016, we sampled caterpillars from eight sites: three sites using *C*. *glabra*, three sites using *P*. *lanceolata*, and two sites using both plants. In 2017, caterpillars were again collected from eight sites: two sites using *C*. *glabra*, three sites using *P*. *lanceolata*, and three sites using both. We did not collect caterpillars at three previously sampled sites in 2017 due to relatively small population sizes, and three additional sites were visited and sampled in 2017 only (Figure 1a). At each site, post‐diapause (fifth or sixth instar) larvae were collected and placed alive into individual, sterile culture tubes (USA Scientific) with foliage from nearby host plants (2016: *n* = 390; 2017: *n* = 229; see Table S1 for *n* at each site). At sites where both host plants were utilized, plant species occurred in discrete spatial patches and larvae were collected based on observed host plant use at the time of collection.

### 2.4 Caterpillar rearing

Field‐collected caterpillars were reared in an incubator at the University of Nevada, Reno using a 16‐hour photoperiod (day temperature: 25°C, night temperature: 20°C) and fed daily with foliage corresponding to their host plant use at the time of collection. *Plantago lanceolata* leaves were collected from the wild in Reno, NV, while *C*. *glabra* leaves were collected from sampling sites in Montpelier, VT and stored in a refrigerator. Leaf surfaces were sterilized prior to feeding by soaking in 5% bleach solution for 10 minutes and rinsing thoroughly. Caterpillars were reared in individual 2 oz. plastic cups and checked daily to monitor development and mortality. Sterile technique was used between handling of each individual, which entailed soaking instruments in a 30% solution of bleach, followed by a 70% solution of ethanol.

Upon reaching the sixth instar and a threshold mass of 0.18 g, caterpillars underwent immune assays (below). Individuals that died prior to reaching this stage (*n* = 148 across both years) were not included in immunological assessments. Following immune assays, caterpillars were either frozen (2016) or returned to the incubator to complete development (2017). Pupated individuals were weighed and transferred to 32 oz. plastic containers with mesh lids for eclosion, and butterflies were maintained on a diet of 10% honey water and monitored daily for mortality.

### 2.5 Caterpillar immune assays

To evaluate the immunocompetence of caterpillars utilizing different host plants, we measured a combination of humoral and cellular immune parameters, including phenoloxidase (hereafter, PO) enzymatic activity, hemocyte concentrations, and melanization. In insects, PO initiates the melanization response, which involves deposition of melanin on the surface of foreign invaders and generation of cytotoxic compounds that contribute to parasite killing (González‐Santoyo & Córdoba‐Aguilar, 2012). The PO cascade and its reactive products have been shown to contribute to antiviral defense in certain insect systems (Shelby & Popham, 2006; Zhao et al., 2011; but see Saejeng et al., 2010; Shikano et al., 2010). Differentiated hemocytes (including granulocytes, oenocytoids, and plasmatocytes) are important mediators of phagocytosis, melanization, and encapsulation (Lavine & Strand, 2002) and can suppress viral infection in Lepidoptera through encapsulation of infected tissues (McNeil et al., 2010; Washburn et al., 1996) and elimination of virus from the hemolymph (Trudeau et al., 2001).

To measure standing PO activity, hemolymph was extracted from caterpillars (*n* = 454) and a spectrophotometric assay of enzymatic activity was immediately performed following the protocol of Smilanich et al. (2018). Hemolymph was extracted by piercing the cuticle of the A6 segment using a fine needle sterilized with 70% ethanol. Using a pipette, 10 µl of hemolymph was added to 500 µl of cold phosphate‐buffered saline (PBS; Sigma‐Aldrich) and vortexed. To provide a substrate for the reaction, 200 µl of l‐Dopa solution (4.0 mM; Sigma‐Aldrich) was added to 100 µl of each PBS‐bound hemolymph sample in a 96‐well microplate (Bio‐Rad). Colorimetric measurements were recorded using an iMark Microplate Absorbance Reader (Bio‐Rad), which measured absorbance at 490 nm every 30 s for 45 min. PO activity was calculated as the slope (OD_490_/min) over the entire 45‐min period, during which time the enzymatic reaction remained in the linear phase. PO activity measurements with values less than zero (*n* = 2) were excluded from analyses.

To estimate hemocyte concentrations, an additional sample of hemolymph (4–10 µl) was extracted from each caterpillar (*n* = 446) and mixed with twice the volume of cold anticoagulant, which was prepared by mixing 0.684 g of EDTA, 0.346 g of citric acid, and 180 ml of PBS and adjusting the pH to 7.4 before each use (Triggs & Knell, 2012). Hemolymph mixtures were refrigerated and examined within 24 h of extraction by pipetting a 10 µl aliquot of each sample into a Neubauer Bright‐Line hemocytometer (Sigma‐Aldrich). Cells falling within the central grid were counted using a compound microscope at 400× magnification, and hemocytes were distinguished as granulocytes, oenocytoids, or plasmatocytes based on morphology when possible (Ribeiro & Brehélin, 2006). Cells that were not differentiable as these hemocyte types were included in total hemocyte counts. Hemolymph samples with fewer than two cells visible on the entire grid (*n* = 9) were excluded from analyses due to potential sampling error. Total and differential hemocyte concentrations (cells/ml) were calculated by multiplying each hemocyte count (cells/100 nl) by a factor of 30,000 to account for sample dilution (2:1) and convert units.

To assess the melanization response, we simulated immune challenge using abiotic implants, which can provide effective estimates of insect resistance to pathogens (Rantala & Roff, 2007) and parasitoids (Smilanich, Dyer, & Gentry, 2009). Monofilament implants were made from abraded nylon fishing line (0.2 mm diameter) cut into 2 mm lengths and knotted at one end to facilitate removal (Rantala & Roff, 2007). Implants were sterilized with 70% ethanol and inserted into the larval hemocoel via the abdominal wound created during hemolymph extraction. Caterpillars (*n* = 391) were allowed to react to implants for 24 h, during which time they were maintained without food in order to clear the gut prior to IG quantification (see below). In 2016, implants were stored in 70% ethanol following removal; due to degradation of encapsulating material, measurements from a subset of individuals (*n* = 30) were excluded from analyses. In 2017, implants were stored dry and frozen, which effectively prevented sample degradation. Implants were photographed at 3.2× magnification using a dissecting microscope mounted with a digital camera (Carl Zeiss Discovery V.8, AxioCam Software). Using the “quick selection” tool in Adobe Photoshop CC 2018 (Adobe Systems Inc.), a mean grey value (MGV) was generated for each photographed implant. MGV is a numerical measure of greyness ranging from 0 to 255, where 0 = pure grey and 255 = pure white. For ease of interpretation, MGVs were transformed into a percentage of melanization [1 – (MGV/maximum MGV)]*100 prior to analysis (Smilanich, Dyer, Chambers, et al., 2009).

### 2.6 Iridoid glycoside quantification in caterpillars

To evaluate the effects of host plant use on chemical defense, caterpillars were freeze‐killed and homogenized following implant removal in 2016. Tissues remaining following removal of a small sample for viral screening (see below) were used to quantify IG sequestration (*n* = 276). Larvae that died prior to immune assessment (*n* = 85) were excluded from the analysis, as they were not starved prior to freezing. Aucubin and catalpol concentrations of caterpillar tissues were quantified using gas chromatography following the methods of Bowers and Stamp (1992). Samples were weighed and extracted overnight in 5 ml methanol, then filtered, and the extract evaporated. An internal standard, phenyl β‐D‐glucopyranoside (0.500 mg), was added to each sample, which was then partitioned between water and ether to remove lipids. The ether fraction was discarded and the water fraction, containing IGs and sugars, was evaporated and extracted in 1 ml methanol overnight. A 100 µl aliquot was removed, evaporated, and then derivatized with 100 µl of Tri‐Sil Z (Sigma‐Aldrich). Aliquots were injected into an Agilent 7890A gas chromatograph (Agilent Technologies) equipped with a flame ionization detector (FID) and Agilent DB‐1 column (Bowers & Collinge, 1992; Fajer et al., 1992; Gardner & Stermitz, 1988) calibrated with aucubin and catalpol standards (HP ChemStation software, v. A.03.34). All IG concentrations were corrected for sample mass and are presented as percent dry weight (mg IGs/mg dry weight), with total IG concentrations representing the sum of aucubin and catalpol concentrations in each sample. A subset of caterpillars with zero values for total IG sequestration (*n* = 17) were excluded from analyses as potential sample degradation from over‐homogenization may have led to undetectable amounts of IGs.

### 2.7 Viral screening of caterpillars

To detect and quantify JcDV infection in *E*. *phaeton*, DNA was extracted from an aliquot of homogenized tissue from each freeze‐killed caterpillar in 2016 (*n* = 389) and each caterpillar, pupa, or butterfly that died in the laboratory in 2017 (*n* = 198). For the subset of individuals that were reared to the adult stage, wings were removed prior to homogenization, whereas whole larvae and pupae were used. Total DNA was extracted from each tissue sample ($\bar{x}$ = 16.06 ± 0.25 mg) using Qiagen DNeasy 96 Blood and Tissue Kits, following the Protocol for Purification of Total DNA from Animal Tissues.

Extracted DNA was screened for JcDV using quantitative PCR, with primers specific to the VP4 capsid protein gene of JcDV (Wang et al., 2013) and arthropod 28S rDNA primers as an internal control (Nice et al., 2009). DNA samples were screened in duplicate for both VP4 and 28S using iTaq Universal SYBR Green Supermix (Bio‐Rad) at a total reaction volume of 10 µl. Reactions were run on a Bio‐Rad CFX96 Optics Module with C1000 Thermal Cycler following the protocol of Smilanich et al. (2018) for VP4, with an initial denaturing step at 95°C for 5 min, followed by 45 cycles of: 95°C for 10 s, 60°C for 15 s, and 72°C for 15 s. A modified protocol was used for 28S, with an initial step at 95°C for 5 min, followed by 45 cycles of: 95°C for 10 s, 57°C for 15 s, and 72°C for 15 s. A melt curve from 65°C to 95°C was performed following each reaction to verify amplification of a single product. Relative viral loads were calculated as 2^−ΔCt^ (Schmittgen & Livak, 2008), representing the abundance of the JcDV VP4 gene relative to the abundance of the internal control [Δ*C*_t_ = mean *C*_t_ (threshold cycle) for VP4 – mean *C*_t_ for 28S] and log‐transformed.

### 2.8 Statistical analyses

All statistical analyses were performed in R version 4.0.4 (R Core Team, 2021). Linear mixed‐effects models (LMMs) were fitted with the “nlme” package (Pinheiro et al., 2020) using REML, and generalized linear mixed‐effects models (GLMMs) were fitted with “lme4” (Bates et al., 2015). Fixed effects structures of LMMs and GLMMs were selected using an information theoretic (IT) approach (Burnham & Anderson, 2002): for each analysis, a set of candidate models that included focal predictor variables, along with combinations of potentially influential covariates and two‐way interactions, was specified. Akaike information criteria corrected for small sample sizes (AICc) were compared among candidate model sets using the “MuMIn” package (Bartoń, 2020), and the model with the best fit (i.e., lowest AICc value) was selected. In cases where a simpler or equally simple candidate model received a similarly high level of AICc support to the best‐fit model (ΔAICc < 2), the results of all suitable models are presented, and the models with higher AICc values are referred to as “alternative model structures.” Results of the best‐fit model(s) for each analysis are presented below; for details on candidate models and parameters relevant to model selection, see Tables S2–S6. In addition to IT‐specified fixed effects, random intercepts for sampling sites were included in all models. All LMM residuals were inspected for normality and homoscedasticity; if heteroscedasticity was detected, the varIdent or varExp structures were applied to accommodate variable spread of residuals (Zuur et al., 2009; see Tables S2–S6). Marginal *R* ^2^ values were calculated using Nakagawa's method with the “performance” package (Lüdecke et al., 2020) and post‐hoc examinations of estimated marginal means were performed using the “emmeans” package (Lenth, 2021). For all analyses, statistical significance was assessed using an alpha level of 0.05.

#### 2.8.1 JcDV occurrence in *E. phaeton* populations

The probabilities of detecting JcDV in *E*. *phaeton* individuals using the native or exotic host plant species were examined using GLMMs with a binomial distribution and logit link‐function. Host plant species was included as a fixed effect, with JcDV presence (Y/N) in freeze‐killed caterpillars (2016), or lab‐deceased caterpillars, pupae, and adults (2017), as response variables. For JcDV‐positive individuals, log‐transformed viral loads were then compared using LMMs with host plant species and life stage at the time of death (for 2017 only) as fixed effects.

#### 2.8.2 Host plant effects on immunocompetence and sequestration

**FIGURE 2:**
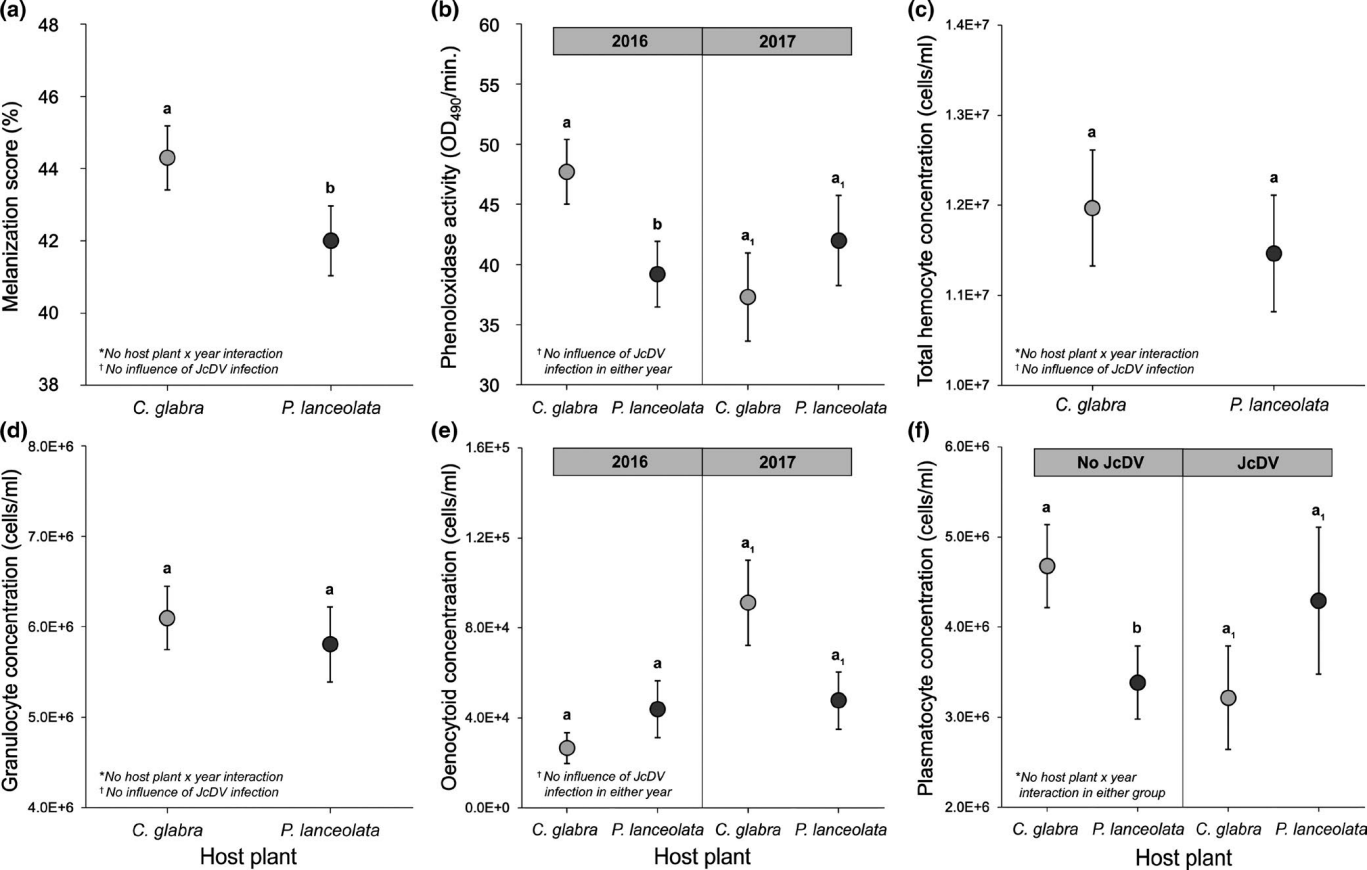
Effects of host plant species on immune responses of *Euphydryas phaeton* caterpillars, including implant melanization score (a), phenoloxidase activity (b), and concentrations of total hemocytes (c), granulocytes (d), oenocytoids (e), and plasmatocytes (f) in the hemolymph. Points represent estimated marginal means (EMMs) ± SE based on linear mixed‐effects models, which included host plant (*Chelone glabra* or *Plantago lanceolata*) and year (2016 or 2017) as fixed effects, larval weight as a covariate, and random intercepts for sites (see Table S7 for full models). EMMs were averaged across larval weights (all models) and years (with the exceptions of b and e, which included significant interactions between host plant and year). The effects of JcDV and its interaction with host plant species were included in only the plasmatocyte model (f). Lettering sets (a–b and a_1_–b_1_) indicate significant differences between estimated marginal means based on pairwise contrasts, evaluated using an alpha level of 0.05. Use of the exotic *P*. *lanceolata* was associated with significant reductions in melanization (a), PO activity (in 2016 only; b), and plasmatocyte concentrations (in uninfected individuals only; f)

Host plant effects on caterpillar immune responses were evaluated separately for each immune parameter using LMMs with host plant, year, and larval weight as fixed effects and PO activity, melanization, and granulocyte, oenocytoid, plasmatocyte, and total hemocyte concentrations as responses. In addition, the interaction between host plant species and year was included in the best‐fit models for PO activity and oenocytoids, and the effects of JcDV infection (Y/N) and its interaction with host plant were included in the best‐fit model for plasmatocytes. These predictors were not retained in the other models, as their inclusion did not improve model fit (Table S3). All hemocyte concentrations were cube‐root transformed, and melanization scores were squared, to improve normality of residuals. The effects of host plant species and significant two‐way interactions are summarized in Figure 2; see Table S7 for full models.

Caterpillar sequestration of aucubin, catalpol, and total IGs was compared across host plant species using separate LMMs including the covariate of larval weight. All IG concentrations were cube‐root transformed to improve normality of residuals. Relationships between IG sequestration and larval immunity were assessed using separate LMMs including the total concentration of sequestered IGs, the composition of sequestered IGs (quantified as the proportion of aucubin out of total IGs), host plant, and influential two‐way interactions (see Table S5) as fixed effects and PO, melanization, and total hemocytes as responses. Follow‐up LMMs evaluated the effects of total IG concentration on each type of hemocytes (granulocytes, oenocytoids, and plasmatocytes).

#### 2.8.3 Effects of immunity and sequestration on viral infection

The effect of sequestration on viral burden was investigated using an LMM with total IG sequestration and host plant species as fixed effects and larval JcDV load as the response. As granulocytes were the primary hemocyte type impacted by sequestration (see Section 3), we then performed an LMM with total IG sequestration, granulocyte concentration, and the interaction between IG sequestration and granulocytes as fixed effects, and JcDV load as the response. To probe this interaction, Johnson–Neyman intervals for significance of the conditional effect of granulocytes on JcDV load were calculated using the “interactions” package (Long, 2019).

Survivorship of JcDV‐infected individuals in 2017 was assessed using two GLMMs with survival to the adult stage (Y/N) as the binomial response variable. The first model evaluated load‐dependent effects on survival, with JcDV load and host plant as fixed effects, while the second examined the effects of larval immune responses (along with larval weight) on survival.

## 3 RESULTS

### 3.1 Occurrence of JcDV in *E. phaeton* populations

JcDV was detected in *E*. *phaeton* individuals originating from all sites, at overall frequencies of 12% in 2016 and 25% in 2017 (see Figure 1b,c for site‐level frequencies). In 2016, viral frequency did not differ significantly between caterpillars utilizing the two host plant species, though the odds of viral detection were slightly higher in individuals using the exotic *P*. *lanceolata* than those using the native *C*. *glabra* [odds ratio (OR) = 1.50, 95% confidence interval (CI) = (0.79–2.85), *p* = .2]. However, caterpillars using *P*. *lanceolata* harbored significantly higher JcDV loads than those using *C*. *glabra* (Figure 1b; *β* = 0.81 ± 0.31, *t* = 2.6, *df* = 34, *p* = .01; marginal *R* ^2^ = .14, *n* = 43), with 138‐fold greater untransformed loads.

In 2017, when individuals were reared out to adulthood or death before viral screening, neither JcDV frequency [OR = 0.64, 95% CI = (0.32–1.38), *p* = .2] nor postmortem loads (*β* = −0.61 ± 0.38, *t* = −1.6, *df* = 36, *p* = .1) differed based on host plant (Figure 1c). However, viral loads were higher in deceased pupae (*β* = 1.57 ± 0.42, *t* = 3.8, *df* = 36, *p* < .001) and larvae (*β* = 2.19 ± 0.63, *t* = 3.5, *df* = 36, *p* = .001) than in butterflies (marginal *R* ^2^ = .35, *n* = 47).

### 3.2 Does use of an exotic host plant impact immunocompetence?

Use of the exotic plant, *P*. *lanceolata*, was associated with a significant reduction in the melanization response of *E*. *phaeton* caterpillars, compared to *C*. *glabra* (Figure 2a; *β* = −190 ± 86, *t* = −2.3, *df* = 377, *p* = .03). PO activity was also reduced in larvae using *P*. *lanceolata* in 2016 but did not differ significantly based on host plant in 2017 (Figure 2b; host plant × year interaction: *β* = 13.2 ± 5.7, *t* = 2.3, *df* = 439, *p* = .02). An alternative model structure that excluded the interaction between host plant and year showed no significant effect of host plant species on PO activity (*β* = −4.0 ± 3.3, *t* = −1.2, *df* = 440, *p* = .2) but had lower support (Table S7).

Total hemocyte concentrations did not vary based on host plant (Figure 2c; *β* = −3.3 ± 5.5, *t* = −0.6, *df* = 432, *p* = .6); however, interesting patterns emerged when investigating different types of hemocytes. Specifically, using *P*. *lanceolata* had a negative impact on plasmatocytes in uninfected caterpillars, but the opposite pattern was observed when caterpillars were infected with JcDV (Figure 2f; host plant × JcDV interaction: *β* = 32 ± 14, *t* = 2.3, *df* = 277, *p* = .03), suggesting that JcDV infection mediated host plant effects on plasmatocyte concentrations. An alternative model structure that excluded the effect of JcDV infection showed no significant effect of host plant species on plasmatocytes (*β* = −10.0 ± 6.8, *t* = −1.5, *df* = 279, *p* = .1) but had lower support (Table S7). Granulocyte concentrations did not differ based on host plant (Figure 2d; *β* = −3.0 ± 5.6, *t* = −0.53, *df* = 292, *p* = .6), while oenocytoid concentrations were marginally lower on *P*. *lanceolata* than *C*. *glabra* in 2017 but not in 2016 (Figure 2e; host plant × year interaction: *β* = −14.1 ± 6.0, *t* = −2.4, *df* =307, *p* = .02). The effect of JcDV infection was not retained in the models for melanization, PO activity, total hemocytes, granulocytes, or oenocytoids, as its inclusion did not improve model fit (Table S3).

### 3.3 Does use of an exotic host plant impact sequestration?

**FIGURE 3:**
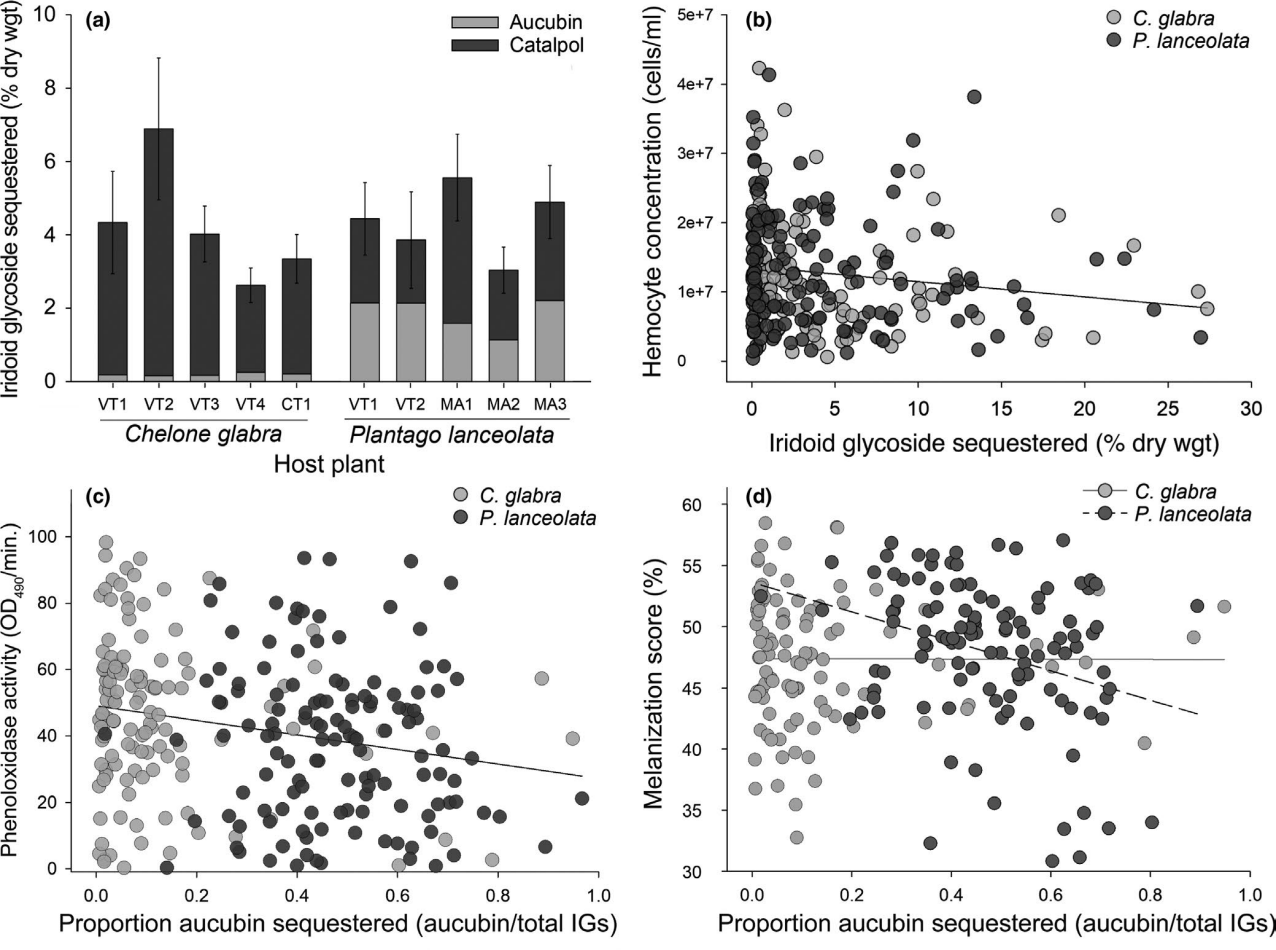
(a) Iridoid glycoside sequestration of *Euphydryas phaeton* caterpillars utilizing either *Chelone glabra* or *Plantago lanceolata* across sites. Bars represent mean total IG concentrations (% dry weight) ± SE, delineated by proportions of aucubin and catalpol (*n* = 7–36 per bar). (b) Relationship between IG sequestration and total hemocyte concentration in *E*. *phaeton* larvae (untransformed values). Caterpillars that sequestered higher concentrations of IGs exhibited significantly lower hemocyte densities, regardless of host plant species (LMM: marginal *R* ^2^ = .04, *n* = 250). (c) Relationship between the proportion of aucubin sequestered by larvae (aucubin/total IGs) and phenoloxidase activity. Caterpillars that sequestered a higher proportion of aucubin exhibited significantly lower PO activity on both host plants (LMM: marginal *R* ^2^ = 0.06, *n* = 252). (d) Relationship between the proportion of aucubin sequestered by larvae and implant melanization. Larvae that sequestered higher proportions of aucubin exhibited significantly lower melanization on the exotic plant, *P*. *lanceolata*, but not the native *C*. *glabra* (LMM: marginal *R* ^2^ = .06, *n* = 219). Significance was assessed using an alpha level of 0.05

*Euphydryas phaeton* caterpillars exhibited distinct patterns of IG sequestration when feeding on *C*. *glabra* and *P*. *lanceolata*, mirroring the typical IG profiles of their respective host plant species (Bowers & Stamp, 1992; Bowers et al., 1992; Duff et al., 1965; Fajer, 1989). While larvae consuming *C*. *glabra* primarily sequestered catalpol, with little to no aucubin, larvae consuming *P*. *lanceolata* sequestered a more even mixture of catalpol and aucubin (Figure 3a). Overall, the total concentration of IGs sequestered by larvae did not differ between the two host plants (*β* = 0.039 ± 0.076, *t* = 0.51, *df* = 266, *p* = .6; marginal *R* ^2^ = .09; *n* = 276). However, caterpillars using *P*. *lanceolata* sequestered over seven times more aucubin than those using *C*. *glabra* (*β* = 0.469 ± 0.044, *t* = 11, *df* = 249, *p* < .001; marginal *R* ^2^ = .73, *n* = 260), while caterpillars using *C*. *glabra* sequestered 42% more catalpol than those using *P*. *lanceolata* (*β* = −0.152 ± 0.074, *t* = −2.1, *df* = 258, *p* = .04; marginal *R* ^2^ = .09, *n* = 268).

### 3.4 Is higher sequestration associated with reduced immunocompetence?

There was a significant negative relationship between the total concentration of IGs sequestered by *E*. *phaeton* larvae and total hemocyte concentration in the hemolymph (Figure 3B; *β* = −13.0 ± 5.4, *t* = −2.4, *df* = 238, *p* = .02). This pattern was evident in individuals utilizing both *C*. *glabra* and *P*. *lanceolata* and was consistent across alternative model structures that varied in their inclusion of two‐way interaction terms (Table S8). In addition, there was no significant relationship between the composition of sequestered IGs (proportion of aucubin) and total hemocytes (*β* = 47 ± 25, *t* = 1.9, *df* = 238, *p* = .06). Follow‐up analysis of the relationships between sequestration and different types of hemocytes revealed that this pattern was mediated by significant negative associations between IG concentration and granulocytes (*β* = −18.4 ± 6.2, *t* = −3.0, *df* = 138, *p* = .004) and IG concentration and plasmatocytes (*β* = −14.6 ± 5.8, *t* = −2.5, *df* = 146, *p* = .01; Table S9).

The concentration of IGs sequestered by caterpillars was not significantly associated with PO activity (*β* = −3.4 ± 2.5, *t* = −1.4, *df* = 241, *p* = .2) or melanization (*β* = 67 ± 99, *t* = 0.68, *df* = 206, *p* = .5); however, compositional variation in IG sequestration did exhibit correlations with these parameters. There was a significant negative relationship between the proportion of aucubin sequestered by caterpillars (aucubin/total IGs) and PO activity on both plants (Figure 3c; *β* = −26.3 ± 9.4, *t* = −2.8, *df* = 241, *p* = .006). A similar pattern was documented for melanization: sequestration of a greater proportion of aucubin was associated with a significant reduction in the melanization response of caterpillars utilizing *P*. *lanceolata*, but not *C*. *glabra* (Figure 3d; IG composition × host plant interaction: *β* = −1203 ± 460, *t* = −2.6, *df* = 206, *p* = .009). This pattern was consistent in an alternative model structure (see Table S8 for full models).

### 3.5 Do host plant effects on sequestration and/or immunocompetence affect interactions with a pathogen?

**FIGURE 4:**
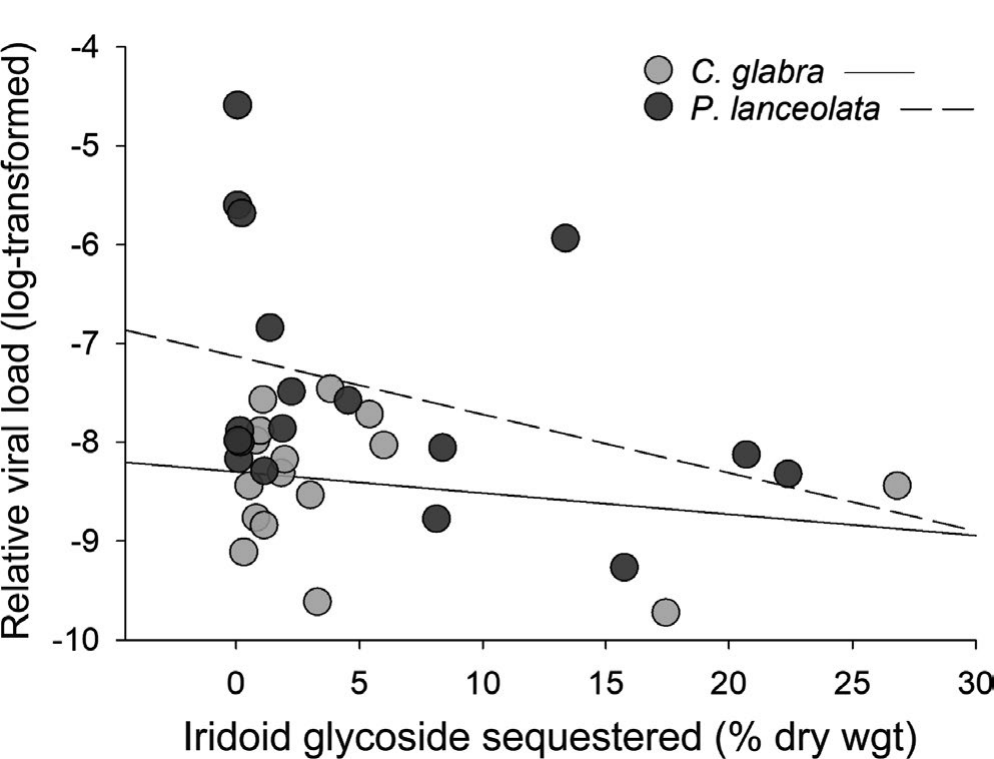
Relationship between iridoid glycoside sequestration and Junonia coenia densovirus load of *Euphydryas phaeton* caterpillars. Based on a linear mixed‐effects model with an alpha level of 0.05, JcDV‐infected caterpillars that sequestered higher total concentrations of IGs exhibited significantly lower viral infection loads when utilizing both the native plant, *Chelone glabra*, and the exotic plant, *Plantago lanceolata* (marginal *R* ^2^ = .26, *n* = 34). Though the negative effect of IG sequestration on viral burden did not differ significantly based on host plant species, separate slopes are provided for individuals using *C*. *glabra* and *P*. *lanceolata* for illustrative purposes

Increased IG sequestration was associated with reduced JcDV load in *E*. *phaeton* larvae using both *P*. *lanceolata* and *C*. *glabra* (Figure 4; *β* = −0.46 ± 0.22, *t* = −2.1, *df* = 24, *p* = .04). Though the interaction between host plant and IG sequestration was not significant (*β* = −0.41 ± 0.47, *t* = −0.89, *df* = 23, *p* = .4), the magnitude of this relationship was greater for caterpillars using *P*. *lanceolata* (slope: −0.59 ± 0.26 for *P*. *lanceolata*; slope: −0.18 ± 0.38 for *C*. *glabra*). Since granulocyte concentrations were negatively associated with sequestration (see above), we examined the interacting influences of sequestration and granulocytes on viral load in the subset of larvae for which all three parameters were quantified. Interestingly, JcDV load was negatively associated with both total IG sequestration (*β* = −4.7 ± 1.1, *t* = −4.4, *df* = 6, *p* = .005) and granulocyte concentration (*β* = −0.0411 ± 0.0090, *t* = −4.6, *df* = 6, *p* = .004). Moreover, there was a significant interaction between sequestration and granulocyte concentration (*β* = 0.0214 ± 0.0064, *t* = 3.3, *df* = 6, *p* = .02; marginal *R* ^2^ = .67, *n* = 16). This interaction indicates that when larvae sequestered IGs at low levels, granulocytes had a significant negative effect on viral load; however, this effect attenuated as caterpillars sequestered higher IG concentrations. The threshold or tipping point at which IG sequestration began to erode the putative immunological effect of granulocytes on JcDV (Johnson–Neyman interval: transformed IG sequestration value <1.5) corresponded to an untransformed value of 3.1% sequestered IGs by dry weight. Above this value, granulocyte concentrations no longer exhibited a significant negative relationship with JcDV burden.

**FIGURE 5:**
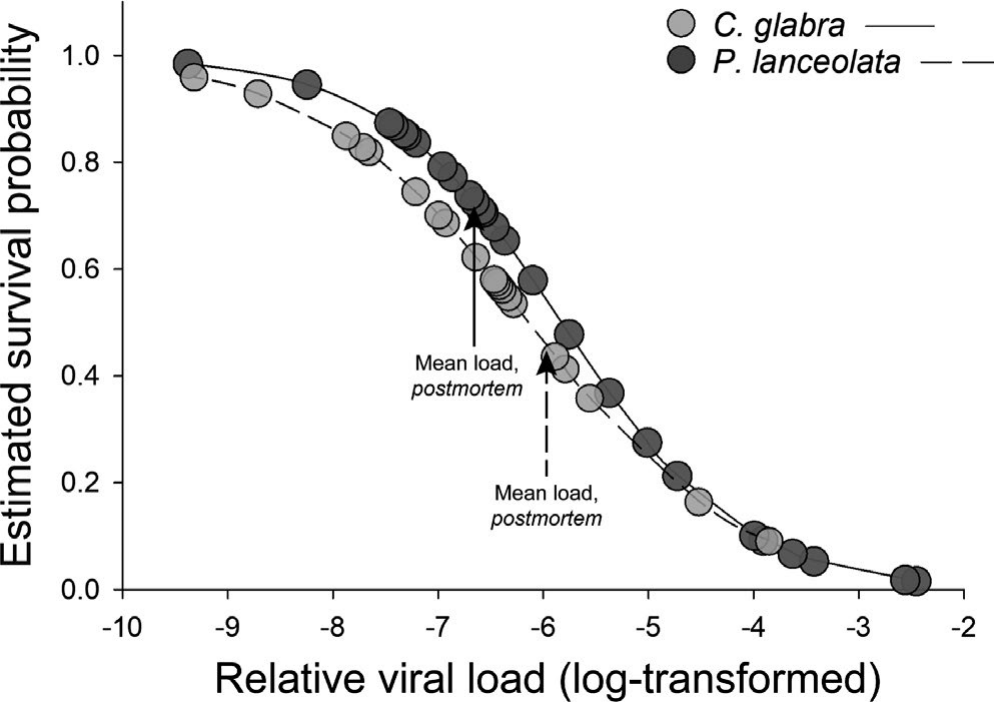
Estimated survival probabilities of *Euphydryas phaeton* based on Junonia coenia densovirus load. Based on a generalized linear mixed‐effects models with an alpha level of 0.05, higher JcDV infection loads corresponded to a lower probability of surviving to the adult stage, regardless of larval host plant species (GLMM: marginal *R* ^2^ = .47, *n* = 47). Arrows indicate mean postmortem viral loads of field‐collected insects in 2017 (reared out and deceased in the laboratory as larvae, pupae, or adults), which did not differ significantly between individuals reared on the native host plant, *Chelone glabra*, and the exotic host plant, *Plantago lanceolata*

Survival of JcDV‐infected individuals was not host plant specific: 46% of infected individuals reared on *C*. *glabra* (*n* = 43) versus 45% of those reared on *P*. *lanceolata* (*n* = 47) died before reaching the adult stage. However, JcDV exhibited a load‐dependent effect on survival: insects with higher viral loads had a lower probability of surviving to the adult stage [Figure 5; OR = 0.32, 95% CI = (0.14–0.50), *p* = .002]. Notably, the strength of larval immune responses did not significantly impact the survival of infected individuals (Table S10).

## 4 DISCUSSION

This study provides evidence that host range expansion mediates a multifaceted shift in herbivore physiological defenses against natural enemies. Use of the exotic host plant, *P*. *lanceolata*, was associated with: (1) suppression of multiple immune parameters (Figure 2), which may influence vulnerability to a broad range of pathogens and parasites and (2) differential composition of sequestered IGs (Figure 3a), which may compromise the efficacy of chemical defense against predators (Bowers, 1980) and impact the strength of immunological defenses against pathogens and parasites (Figure 3c,d). Additionally, in situ JcDV infection loads were higher on *P*. *lanceolata* (Figure 1b), suggesting that use of the exotic plant may entail greater exposure or vulnerability to this pathogen during larval development. Despite these defensive differences, larvae using both *P*. *lanceolata* and *C*. *glabra* exhibited reduced JcDV burdens when sequestering high concentrations of IGs (Figure 4), and survival of JcDV‐infected individuals was ultimately similar on the two plants (Figure 5). These results indicate that use of an exotic plant is characterized by reduced immunocompetence but comparable levels of chemical protection against a virus, potentially supporting sustainable populations of *E*. *phaeton* on this exotic plant.

Enhancement of immunity does not appear to be promoting *E*. *phaeton* persistence on the exotic plant, as field‐collected caterpillars exhibited reductions in multiple immune responses when consuming *P*. *lanceolata*. The suite of immune parameters suppressed in caterpillars using the exotic plant, including melanization (Figure 2a), plasmatocyte concentration (Figure 2f), and PO activity (in 2016; Figure 2b), provides defense against eukaryotic parasites (Carton et al., 2008; Richman & Kafatos, 1996) and can also contribute to antiviral (McNeil et al., 2010; Trudeau et al., 2001; Washburn et al., 1996) and antimicrobial (Lavine & Strand, 2002; Rantala & Roff, 2007) immunity. In particular, decreased melanization can be an effective predictor of successful parasitism by dipteran and hymenopteran parasitoids in Lepidoptera (Smilanich, Dyer, & Gentry, 2009; but see Klemola et al., 2008), indicating that caterpillars with weaker melanization responses may be more vulnerable to parasitism in the wild. Reduced plasmatocyte densities may additionally impair critical cell‐mediated defenses against microbial pathogens, including phagocytosis and expression of antimicrobial peptides (Strand, 2008). Overall, suppression of humoral and cellular components of the immune response represents a substantial putative cost of exotic host plant use for *E*. *phaeton* larvae; however, the extent to which these patterns are consistent across time warrants consideration. Variation in host plant effects across the two sampling years was evident for two out of six measured immune parameters (PO activity, Figure 2b; and oenocytoid densities, Figure 2e), underscoring the likelihood that aspects of larval history beyond host plant identity, including both abiotic and biotic factors, impact patterns of immunocompetence in wild settings.

Host plant use may additionally impact herbivore defense against natural enemies through effects of phytochemical sequestration on the immune response. We documented a negative relationship between IG sequestration and hemocyte concentrations in *E*. *phaeton* caterpillars (Figure 3b), indicating that sequestration of plant secondary metabolites may suppress components of the cellular immune response (specifically, granulocytes and plasmatocytes; see Table S8). This pattern could be a product of direct cytotoxicity of sequestered IGs (Smilanich, Dyer, Chambers, et al., 2009) or indirect effects arising from the competing energetic demands of sequestration and immunity. These findings provide the first support for the “vulnerable host” hypothesis from a field‐based study, as our results indicate that *E*. *phaeton* larvae that sequester high concentrations of IGs may be more immunologically vulnerable to parasitism and/or infectious disease in the wild (Lampert & Bowers, 2015; Smilanich, Dyer, Chambers, et al., 2009).

Although the total concentration of IGs sequestered by *E*. *phaeton* did not differ based on host plant species, caterpillars consuming the exotic *P*. *lanceolata* sequestered a relatively even mixture of aucubin and catalpol, while those consuming *C*. *glabra* sequestered primarily catalpol (Figure 3a). This compositional difference in IG sequestration may have repercussions for the strength of immune responses: both PO activity (Figure 3c) and melanization (Figure 3d) decreased as caterpillars sequestered a greater proportion of aucubin, relative to catalpol. These findings are consistent with previous research documenting negative synergistic effects of aucubin and catalpol sequestration on melanization in another specialist caterpillar (Richards et al., 2012). Though additional research will be necessary to determine the role of synergy in mediating these patterns in *E*. *phaeton*, these results indicate that the distinct IG mixture sequestered by larvae using *P*. *lanceolata* may contribute to immunosuppression on this plant (Figure 2), highlighting a potential tritrophic outcome of compositional phytochemical variation in this system.

Although individuals that sequestered high levels of IGs were more immunologically vulnerable, our results indicate that sequestered phytochemicals may provide herbivores with an additional form of defense against viral infection (Figure 4). We observed a negative relationship between IG sequestration and JcDV load in *E*. *phaeton* caterpillars using both the exotic and native host plants (Figure 4). This apparently protective effect of IG sequestration was surprising, given the negative relationship between IG sequestration and hemocyte concentrations also documented in this study (Figure 3b), and suggests that these compounds may attenuate viral infection through mechanisms external to the immune responses measured in this study. Antiviral properties of IGs are well‐documented in mammalian systems (e.g., Tundis et al., 2008); thus, sequestration of high concentrations of these compounds could directly interfere with JcDV infectivity or replication within the host. Similar protective effects of toxic phytochemicals against pathogens have been reported in several insect systems (Cory & Hoover, 2006; De Roode et al., 2008; Smilanich et al., 2018). Our data expand upon this research by directly associating variation in *sequestration* of plant secondary metabolites with defense against an entomopathogen, a relationship which has rarely been explicitly assessed.

Together, these results highlight an important caveat to the “vulnerable host” hypothesis: the immune response is not the only form of defense against enemies, and host vulnerability may be influenced by both direct and indirect effects (mediated by the immune response) of sequestered compounds on parasites and pathogens. Although not tested here, the potential effects of IG sequestration may give rise to a tradeoff between chemical and immunological forms of defense against the virus, as high levels of sequestration were associated with both attenuation of infection (Figure 4) and suppression of cellular immune components (Figure 3b). In particular, JcDV loads were negatively associated with granulocyte concentrations, indicating that these cells may play a role in suppressing infection. However, the negative relationship between granulocytes and viral load was only evident in larvae sequestering low concentrations of IGs, further suggesting an incompatibility between sequestration and hemocytic immunity.

This immunological cost of sequestration may be expected to give rise to distinct defensive syndromes across herbivore populations experiencing differential pressure from natural enemies. Intermediate sequestration phenotypes may be favored when chemical and hemocytic defenses are both critical for survival; this may be expected in *E*. *phaeton* populations where both JcDV infection (Figure 1) and attack by other natural enemies, including parasitoids and microbial pathogens, are common. Alternatively, chemical defense may be favored over hemocytic immunity in cases where: (1) sequestration provides comparable protection against enemies, but incurs a lesser energetic cost, or (2) sequestration provides defense against a broader suite of enemies, including predators, parasitoids, and pathogens. In such cases, we may predict a disinvestment in immunity (see Tan et al., 2019). Variation in selective pressures from multiple types of natural enemies across *E*. *phaeton* populations using either *C*. *glabra* or *P*. *lanceolata*, and its role in driving tradeoffs in defensive strategies, warrants further study.

Our understanding of the ecological factors facilitating herbivore persistence on exotic host plants may be improved through field‐based investigations within a tritrophic framework. Though the immunological disadvantages of using *P*. *lanceolata* may limit *E*. *phaeton's* ability to persist on this exotic plant, the putatively protective role of IG sequestration against JcDV infection was consistent between host plants. This comparable level of chemical defense may facilitate continued use of the exotic plant, particularly in populations where JcDV pressure is high and mounting an immune response is costly. Importantly, the likelihood of surviving JcDV infection did not differ between individuals reared on the native and exotic host plants (Figure 5), suggesting that chemical defense effectively compensated for immunosuppression on the exotic plant where this pathogen was concerned. These findings provide insight into the paradox of exotic host plant use (Yoon & Read, 2016) in *E*. *phaeton*, indicating that *P*. *lanceolata* may represent an equally suitable host plant, relative to the native *C*. *glabra*, within certain tritrophic contexts. While these defensive traits represent a subset of many dimensions of herbivore fitness that can differ on native and exotic host plants (Forister et al., 2020; Nylin et al., 2018), they provide novel insight into the interacting roles of immune defense and phytochemical sequestration in mediating tritrophic interactions in the wild. Moving forward, consideration of herbivore physiological defenses against higher trophic levels may provide opportunities for more comprehensive evaluation of the ecological costs and benefits of host range expansion, with applications to understanding the evolution of herbivore diet breadth.

## CONFLICT OF INTEREST

The authors declare no competing interests.

## AUTHOR CONTRIBUTIONS

**Nadya D. Muchoney:** Conceptualization (equal); Data curation (lead); Formal analysis (lead); Funding acquisition (supporting); Investigation (lead); Methodology (equal); Project administration (supporting); Software (lead); Supervision (equal); Visualization (equal); Writing – original draft (lead); Writing – review & editing (equal). **M. Deane Bowers:** Conceptualization (equal); Funding acquisition (lead); Methodology (equal); Project administration (lead); Resources (equal); Supervision (equal); Writing – review & editing (equal). **Adrian L. Carper:** Data curation (supporting); Investigation (supporting); Methodology (equal); Project administration (supporting); Supervision (equal); Writing – review & editing (equal). **Peri A. Mason:** Conceptualization (equal); Investigation (supporting); Methodology (equal); Project administration (supporting); Supervision (equal); Writing – review & editing (equal). **Mike B. Teglas:** Funding acquisition (lead); Methodology (equal); Resources (equal); Supervision (equal); Writing – review & editing (equal). **Angela M. Smilanich:** Conceptualization (equal); Funding acquisition (lead); Methodology (equal); Project administration (lead); Resources (equal); Supervision (equal); Visualization (equal); Writing – review & editing (equal).

## Supporting information

Table S1‐S10

## Data Availability

Data supporting the results presented in this study are available in the Dryad Digital Repository (https://doi.org/10.5061/dryad.mkkwh712c).
