# Supplementary material for: Use of an exotic host plant shifts immunity, chemical defense, and viral burden in wild populations of a specialist insect herbivore
Source: Ecol Evol. 2022 Mar 16;12(3):e8723. doi: 10.1002/ece3.8723 (PMC8928866; doi:10.1002/ece3.8723)
Supplement: Supplementary file 1 — Table S1‐S10 [file ECE3-12-e8723-s001.docx]

**APPENDIX**

**Table S1.** Sampling details for *Euphydryas phaeton* caterpillars collected from the wild in May 2016 and 2017. Field sites were located throughout the northeastern U.S. in Connecticut (CT), Massachusetts (MA), Rhode Island (RI), and Vermont (VT), spanning a range of approximately 330 km. At each site, post-diapause *E. phaeton* caterpillars utilized either the native host plant, *Chelone glabra,* the exotic host plant, *Plantago lanceolata,* or both host plants (MA4, VT1, VT2)*.* Sample sizes of caterpillars collected from each site (*n*) are provided for each year.

| **Site ID** | **Host plant species** | **Larvae 2016 (*n*)** | **Larvae 2017 (*n*)** | **Latitude** | | **Longitude** | |
| --- | --- | --- | --- | --- | --- | --- | --- |
| CT1 | *Chelone glabra* | 49 | Not collected* | 41.961877 | | -73.344344 | |
| MA1 | *Plantago lanceolata* | 50 | 30 | 41.636994 | | -70.559876 | |
| MA2 | *Plantago lanceolata* | 50 | 30 | 41.684528 | | -70.400126 | |
| MA3 | *Plantago lanceolata* | 50 | Not collected* | 42.260065 | | -70.877680 | |
| MA4 | *Chelone glabra* | Not visited | 9 | 42.259651 | | -72.092595 | |
| MA4 | *Plantago lanceolata* | Not visited | 12 | 42.259651 | | -72.092595 | |
| RI1 | *Plantago lanceolata* | Not visited | 30 | 41.507292 | | -71.168968 | |
| VT1 | *Chelone glabra* | 25 | 22 | 44.249298 | | -72.513361 | |
| VT1 | *Plantago lanceolata* | 25 | 18 | 44.249298 | | -72.513361 | |
| VT2 | *Chelone glabra* | 25 | 29 | 44.262020 | | -72.505049 | |
| VT2 | *Plantago lanceolata* | 16 | Not collected* | 44.262020 | | -72.505049 | |
| VT3 | *Chelone glabra* | 50 | Not collected* | 44.277640 | | -72.575906 | |
| VT4 | *Chelone glabra* | 50 | 30 | 44.282757 | | -72.542557 | |
| VT5 | *Chelone glabra* | Not visited | 19 | 42.74774 | | -73.073560 | |
| * *Caterpillars not collected due to relatively small population sizes.* | | | | |  |  |  |

| **Model Selection: *Occurrence of JcDV in Euphydryas phaeton populations*** | | | | | | | | | |
| --- | --- | --- | --- | --- | --- | --- | --- | --- | --- |
| *Response variable* | *Year* | *Type* | *n* | *Variance*  *structure* | *Fixed effects structure* | *df* | *logLik* | *AICc* | *weight* |
| Caterpillar JcDV  (presence/absence) | 2016 | GLMM | 389 | None | *Model 1.1:* Host plant | 3 | -112.4 | 230.9 | 0.62 |
|  |  |  |  |  | *Model 1.2:* Host plant + weight | 4 | -112.4 | 232.9 | 0.23 |
|  |  |  |  |  | *Model 1.3:* Host plant + weight + host plant*weight | 5 | -111.8 | 233.8 | 0.15 |
| Caterpillar JcDV load | 2016 | LMM | 43 | None | *Model 2.1:* Host plant | 4 | -51.2 | 111.7 | 0.73 |
|  |  |  |  |  | *Model 2.2:* Host plant + weight | 5 | -51.1 | 114.2 | 0.21 |
|  |  |  |  |  | *Model 2.3:* Host plant + weight + host plant*weight | 6 | -51.0 | 116.8 | 0.06 |
| Deceased JcDV (Y/N) | 2017 | GLMM | 196 | None | *Model 3:* Host plant | *No model selection performed* | | | |
| Deceased JcDV load  (presence/absence) | 2017 | LMM | 47 | None | *Model 4.1:* Host plant | 4 | -86.4 | 181.7 | 0.00 |
|  |  |  |  |  | *Model 4.2:* Host plant + life stage | 6 | -77.0 | 168.1 | 0.79 |
|  |  |  |  |  | *Model 4.3:* Host plant + life stage + host plant*life stage | 8 | -75.5 | 170.7 | 0.21 |

**Table S2.** Details of model selection for linear mixed-effects models (LMMs) and generalized linear mixed-effects models (GLMMs) presented in the Results section: “Occurrence of JcDV in *Euphydryas phaeton* populations.” For each analysis, a set of candidate models was specified, and the degrees of freedom (*df*), log-likelihood (*logLik*), Akaike’s information criterion corrected for small sample sizes (*AICc*), and Akaike weight (*weight*) of each model was calculated. Fixed effects structures were selected using an information theoretic (IT) approach: the model with the best fit (i.e., the lowest AICc value; indicated with shading) was reported in the Results. All models included random intercepts for sampling sites. Host plant = host plant species (*Chelone glabra* or *Plantago lanceolata*); weight = larval body weight at the time of immune assessment; JcDV load = log-normalized JcDV load relative to an internal control gene; deceased = lab-deceased larvae, pupae, and adults; stage = life stage (larva, pupa, or adult) at the time of death.

**Table S3.** Details of model selection for linear mixed-effects models (LMMs) reported in the Results section: “Does use of an exotic host plant impact immunocompetence?” For each analysis, a set of candidate models was specified, and the degrees of freedom (*df*), log-likelihood (*logLik*), Akaike’s information criterion corrected for small sample sizes (*AICc*), and Akaike weight (*weight*) of each model was calculated. Fixed effects structures were selected using an information theoretic (IT) approach: the model with the best fit (i.e., the lowest AICc value; indicated with shading) was reported in the Results. For PO activity and plasmatocytes (candidate model sets 5 and 9), the results of two models were reported, as a simpler model structure that excluded certain fixed effects was found to receive a similarly high level of AICc support to the best-fit model (ΔAICc < 2). All models included random intercepts for sampling sites, and variance structures (varIdent or varExp) were applied to a subset of LMMs to account for heterogeneity of variance across levels of fixed effects. Host plant = host plant species (*Chelone glabra* or *Plantago lanceolata*); year = sampling year (2016 or 2017); weight = larval body weight at the time of immune assessment; JcDV = presence/absence of Junonia coenia densovirus infection.

| **Model Selection: *Does use of an exotic host plant impact immunocompetence?*** | | | | | | | | | | |
| --- | --- | --- | --- | --- | --- | --- | --- | --- | --- | --- |
| *Response variable* | *Year* | *Type* | *n* | *Variance*  *structure* | *Fixed effects structure* | *df* | *logLik* | *AICc* | *weight* |  |
| PO activity | Both | LMM | 454 | varIdent(year) | *Model 5.1:* Host plant + year + weight | 7 | -1991.2 | 3996.7 | 0.17 |  |
|  |  |  |  |  | *Model 5.2:* Host plant + year + weight + JcDV | 8 | -1991.2 | 3998.7 | 0.06 |  |
|  |  |  |  |  | *Model 5.3:* Host plant + year + weight + JcDV+ host plant*JcDV | 9 | -1990.8 | 3999.9 | 0.03 |  |
|  |  |  |  |  | *Model 5.4:* Host plant + year + weight + host plant*year | 8 | -1989.3 | 3994.9 | 0.43 |  |
|  |  |  |  |  | *Model 5.5:* Host plant + year + weight + JcDV+ host plant*year | 9 | -1989.2 | 3996.9 | 0.15 |  |
|  |  |  |  |  | *Model 5.6:* Host plant + year + weight + JcDV+ host plant*year + host plant*JcDV | 10 | -1988.3 | 3997.0 | 0.15 |  |
| Melanization | Both | LMM | 391 | varExp(weight) | *Model 6.1:* Host plant + year + weight | 7 | -2830.7 | 5675.7 | 0.33 |  |
|  |  |  |  |  | *Model 6.2:* Host plant + year + weight + JcDV | 8 | -2829.8 | 5676.1 | 0.28 |  |
|  |  |  |  |  | *Model 6.3:* Host plant + year + weight + JcDV+ host plant*JcDV | 9 | -2829.8 | 5678.1 | 0.10 |  |
|  |  |  |  |  | *Model 6.4:* Host plant + year + weight + host plant*year | 8 | -2830.6 | 5677.5 | 0.13 |  |
|  |  |  |  |  | *Model 6.5:* Host plant + year + weight + JcDV+ host plant*year | 9 | -2829.6 | 5677.8 | 0.12 |  |
|  |  |  |  |  | *Model 6.6:* Host plant + year + weight + JcDV+ host plant*year + host plant*JcDV | 10 | -2829.6 | 5679.9 | 0.04 |  |
| Granulocytes | Both | LMM | 306 | None | *Model 7.1:* Host plant + year + weight | 6 | -1488.2 | 2988.6 | 0.45 |  |
|  |  |  |  |  | *Model 7.2:* Host plant + year + weight + JcDV | 7 | -1488.1 | 2990.7 | 0.16 |  |
|  |  |  |  |  | *Model 7.3:* Host plant + year + weight + JcDV+ host plant*JcDV | 8 | -1487.7 | 2991.9 | 0.09 |  |
|  |  |  |  |  | *Model 7.4:* Host plant + year + weight + host plant*year | 7 | -1487.9 | 2990.3 | 0.20 |  |
|  |  |  |  |  | *Model 7.5:* Host plant + year + weight + JcDV+ host plant*year | 8 | -1487.9 | 2992.4 | 0.07 |  |
|  |  |  |  |  | *Model 7.6:* Host plant + year + weight + JcDV+ host plant*year + host plant*JcDV | 9 | -1487.6 | 2993.8 | 0.03 |  |
| Oenocytoids | Both | LMM | 322 | None | *Model 8.1:* Host plant + year + weight | 6 | -1391.4 | 2795.1 | 0.08 |  |
|  |  |  |  |  | *Model 8.2:* Host plant + year + weight + JcDV | 7 | -1391.2 | 2796.9 | 0.03 |  |
|  |  |  |  |  | *Model 8.3:* Host plant + year + weight + JcDV+ host plant*JcDV | 8 | -1390.5 | 2797.5 | 0.02 |  |
|  |  |  |  |  | *Model 8.4:* Host plant + year + weight + host plant*year | 7 | -1388.8 | 2792.0 | 0.37 |  |
|  |  |  |  |  | *Model 8.5:* Host plant + year + weight + JcDV+ host plant*year | 8 | -1388.7 | 2793.9 | 0.15 |  |
|  |  |  |  |  | *Model 8.6:* Host plant + year + weight + JcDV+ host plant*year + host plant*JcDV | 9 | -1386.8 | 2792.2 | 0.34 |  |
| Plasmatocytes | Both | LMM | 293 | None | *Model 9.1:* Host plant + year + weight | 6 | -1545.6 | 3103.4 | 0.18 |  |
|  |  |  |  |  | *Model 9.2:* Host plant + year + weight + JcDV | 7 | -1545.2 | 3104.8 | 0.09 |  |
|  |  |  |  |  | *Model 9.3:* Host plant + year + weight + JcDV+ host plant*JcDV | 8 | -1542.7 | 3101.9 | 0.38 |  |
|  |  |  |  |  | *Model 9.4:* Host plant + year + weight + host plant*year | 7 | -1544.8 | 3104.0 | 0.13 |  |
|  |  |  |  |  | *Model 9.5:* Host plant + year + weight + JcDV+ host plant*year | 8 | -1544.5 | 3105.5 | 0.06 |  |
|  |  |  |  |  | *Model 9.6:* Host plant + year + weight + JcDV+ host plant*year + host plant*JcDV | 9 | -1542.5 | 3103.6 | 0.16 |  |
| Total hemocytes | Both | LMM | 446 | varExp(weight) | *Model 10.1:* Host plant + year + weight | 7 | -2254.5 | 4523.2 | 0.44 |  |
|  |  |  |  |  | *Model 10.2:* Host plant + year + weight + JcDV | 8 | -2254.4 | 4525.2 | 0.16 |  |
|  |  |  |  |  | *Model 10.3:* Host plant + year + weight + JcDV+ host plant*JcDV | 9 | -2253.9 | 4526.3 | 0.10 |  |
|  |  |  |  |  | *Model 10.4:* Host plant + year + weight + host plant*year | 8 | -2254.3 | 4525.0 | 0.16 |  |
|  |  |  |  |  | *Model 10.5:* Host plant + year + weight + JcDV+ host plant*year | 9 | -2254.3 | 4527.0 | 0.07 |  |
|  |  |  |  |  | *Model 10.6:* Host plant + year + weight + JcDV+ host plant*year + host plant*JcDV | 10 | -2253.6 | 4527.7 | 0.05 |  |

| **Model Selection:**  ***Does use of an exotic host plant impact sequestration?*** | | | | | | | | | |
| --- | --- | --- | --- | --- | --- | --- | --- | --- | --- |
| *Response variable* | *Year* | *Type* | *n* | *Variance*  *structure* | *Fixed effects structure* | *df* | *logLik* | *AICc* | *weight* |
| IG concentration | 2016 | LMM | 276 | None | *Model 11.1:* Host plant | 4 | -273.2 | 554.5 | 0.00 |
|  |  |  |  |  | *Model 11.2:* Host plant + weight | 5 | -260.7 | 532.0 | 0.71 |
|  |  |  |  |  | *Model 11.3:* Host plant + weight + host plant*weight | 6 | -260.9 | 533.7 | 0.29 |
| Aucubin concentration | 2016 | LMM | 260 | varIdent (host plant) | *Model 12.1:* Host plant | 5 | -45.2 | 100.7 | 0.00 |
|  |  |  |  |  | *Model 12.2:* Host plant + weight | 6 | -39.6 | 91.6 | 0.02 |
|  |  |  |  |  | *Model 12.3:* Host plant + weight + host plant*weight | 7 | -34.8 | 84.0 | 0.98 |
| Catalpol concentration | 2016 | LMM | 268 | None | *Model 13.1:* Host plant | 4 | -250.8 | 509.8 | 0.00 |
|  |  |  |  |  | *Model 13.2:* Host plant + weight | 5 | -241.3 | 492.8 | 0.50 |
|  |  |  |  |  | *Model 13.3:* Host plant + weight + host plant*weight | 6 | -240.3 | 492.9 | 0.50 |

**Table S4.** Details of model selection for linear mixed-effects models (LMMs) reported in the Results section: “Does use of an exotic host plant impact sequestration?” For each analysis, a set of candidate models was specified, and the degrees of freedom (*df*), log-likelihood (*logLik*), Akaike’s information criterion corrected for small sample sizes (*AICc*), and Akaike weight (*weight*) of each model was calculated. Fixed effects structures were selected using an information theoretic (IT) approach: the model with the best fit (i.e., the lowest AICc value; indicated with shading) was reported in the Results. All models included random intercepts for sampling sites, and variance structures (varIdent or varExp) were applied to a subset of LMMs to account for heterogeneity of variance across levels of fixed effects. IG concentration = total concentration of iridoid glycosides sequestered by *Euphydryas phaeton* caterpillars; host plant = host plant species (*Chelone glabra* or *Plantago lanceolata*); weight = larval body weight at the time of immune assessment.

**Table S5.** Details of model selection for linear mixed-effects models (LMMs) reported in the Results section: “Is higher sequestration associated with reduced immunocompetence?” For each analysis, a set of candidate models was specified, and the degrees of freedom (*df*), log-likelihood (*logLik*), Akaike’s information criterion corrected for small sample sizes (*AICc*), and Akaike weight (*weight*) of each model was calculated. Fixed effects structures were selected using an information theoretic (IT) approach: the model with the best fit (i.e., the lowest AICc value; indicated with shading) was reported in the Results. For melanization and total hemocytes (model sets 15 and 16), the results of 2-3 models were reported, as model structures that included an equal or lesser number of fixed effects were found to receive a similarly high level of AICc support to the best-fit model (ΔAICc < 2). All models included random intercepts for sampling sites, and the varIdent variance structure was applied to one LMM to account for heterogeneity of variance. Host plant = host plant species (*Chelone glabra* or *Plantago lanceolata*); IG concentration = total concentration of iridoid glycosides sequestered by *Euphydryas phaeton* caterpillars; IG composition = proportion of aucubin sequestered out of total IGs.

| **Model Selection: *Is higher sequestration associated with reduced immunocompetence?*** | | | | | | | | | |
| --- | --- | --- | --- | --- | --- | --- | --- | --- | --- |
| *Response variable* | *Year* | *Type* | *n* | *Variance*  *structure* | *Fixed effects structure* | *df* | *logLik* | *AICc* | *weight* |
| PO activity | 2016 | LMM | 252 | None | *Model 14.1:* Host plant + IG concentration + IG composition | 6 | -1147.0 | 2306.3 | 0.53 |
|  |  |  |  |  | *Model 14.2:* Host plant + IG concentration + IG composition + host plant*IG concentration | 7 | -1146.9 | 2308.3 | 0.19 |
|  |  |  |  |  | *Model 14.3:* Host plant + IG concentration + IG composition + host plant*IG composition | 7 | -1146.9 | 2308.2 | 0.20 |
|  |  |  |  |  | *Model 14.4:* Host plant + IG concentration + IG composition + host plant*IG concentration   + host plant*IG composition | 8 | -1146.8 | 2310.2 | 0.08 |
| Melanization | 2016 | LMM | 219 | None | *Model 15.1:* Host plant + IG concentration + IG composition | 6 | -1675.1 | 3362.6 | 0.10 |
|  |  |  |  |  | *Model 15.2:* Host plant + IG concentration + IG composition + host plant*IG concentration | 7 | -1675.0 | 3364.6 | 0.04 |
|  |  |  |  |  | *Model 15.3:* Host plant + IG concentration + IG composition + host plant*IG composition | 7 | -1672.6 | 3359.7 | 0.41 |
|  |  |  |  |  | *Model 15.4:* Host plant + IG concentration + IG composition + host plant*IG concentration   + host plant*IG composition | 8 | -1671.4 | 3359.5 | 0.45 |
| Total hemocytes | 2016 | LMM | 250 | varIdent (host plant) | *Model 16.1:* Host plant + IG concentration + IG composition | 7 | -1329.0 | 2672.5 | 0.26 |
|  |  |  |  |  | *Model 16.2:* Host plant + IG concentration + IG composition + host plant*IG concentration | 8 | -1327.8 | 2672.3 | 0.28 |
|  |  |  |  |  | *Model 16.3:* Host plant + IG concentration + IG composition + host plant*IG composition | 8 | -1327.8 | 2672.1 | 0.30 |
|  |  |  |  |  | *Model 16.4:* Host plant + IG concentration + IG composition + host plant*IG concentration   + host plant*IG composition | 9 | -1327.3 | 2673.3 | 0.16 |
| Granulocytes | 2016 | LMM | 148 | None | *Model 17.1:* Host plant + IG concentration | 5 | -773.4 | 1557.2 | 0.67 |
|  |  |  |  |  | *Model 17.2:* Host plant + IG concentration + host plant*IG concentration | 6 | -773.0 | 1558.6 | 0.33 |
| Oenocyoids | 2016 | LMM | 165 | None | *Model 18.1:* Host plant + IG concentration | 5 | -766.8 | 1544.1 | 0.60 |
|  |  |  |  |  | *Model 18.2:* Host plant + IG concentration + host plant*IG concentration | 6 | -766.2 | 1544.9 | 0.40 |
| Plasmatocytes | 2016 | LMM | 156 | None | *Model 19.1:* Host plant + IG concentration | 5 | -814.8 | 1640.0 | 0.74 |
|  |  |  |  |  | *Model 19.2:* Host plant + IG concentration + host plant*IG concentration | 6 | -814.7 | 1642.0 | 0.26 |

**Table S6.** Details of model selection for linear mixed-effects models (LMMs) reported in the Results section: “Do host plant effects on sequestration and/or immunocompetence affect interactions with a pathogen?” For each analysis, a set of candidate models was specified, and the degrees of freedom (*df*), log-likelihood (*logLik*), Akaike’s information criterion corrected for small sample sizes (*AICc*), and Akaike weight (*weight*) of each model was calculated. Fixed effects structures were selected using an information theoretic (IT) approach: the model with the best fit (i.e., the lowest AICc value; indicated with shading) was reported in the Results. All models included random intercepts for sampling sites. IG concentration = total concentration of iridoid glycosides sequestered by *Euphydryas phaeton* caterpillars; IG composition = proportion of aucubin sequestered out of total IGs; host plant = host plant species (*Chelone glabra* or *Plantago lanceolata*); weight = larval body weight at the time of immune assessment.

| **Model Selection:**  ***Do host plant effects on sequestration and/or immunocompetence affect interactions with a pathogen?*** | | | | | | | | | | |
| --- | --- | --- | --- | --- | --- | --- | --- | --- | --- | --- |
| *Response variable* | *Year* | *Type* | *n* | *Variance*  *structure* | *Fixed effects structure* | *df* | *logLik* | *AICc* | *weight* |  |
| Caterpillar JcDV load | 2016 | LMM | 34 | None | *Model 20.1:* IG concentration + host plant | 5 | -45.3 | 102.7 | 0.60 |  |
|  |  |  |  |  | *Model 20.2:* IG concentration + host plant + IG concentration*host plant | 6 | -44.9 | 104.8 | 0.21 |  |
|  |  |  |  |  | *Model 20.3:* IG concentration + host plant + weight | 6 | -45.3 | 105.6 | 0.14 |  |
|  |  |  |  |  | *Model 20.4:* IG concentration + host plant + IG concentration*host plant + weight | 7 | -44.8 | 108.0 | 0.04 |  |
| Caterpillar JcDV load | 2016 | LMM | 16 | None | *Model 21.1:* IG concentration + granulocytes | 5 | -16.4 | 48.8 | 0.06 |  |
|  |  |  |  |  | *Model 21.2:* IG concentration + granulocytes + IG concentration*granulocytes | 6 | -11.2 | 43.6 | 0.82 |  |
|  |  |  |  |  | *Model 21.3:* IG concentration + granulocytes + host plant | 6 | -15.0 | 51.4 | 0.02 |  |
|  |  |  |  |  | *Model 21.4:* IG concentration + granulocytes + host plant   + IG concentration*granulocytes | 7 | -9.9 | 47.8 | 0.10 |  |
| Survival to adult (Y/N) | 2017 | GLMM | 47 | None | *Model 22.1:* JcDV load + host plant | 4 | -23.2 | 55.4 | 0.77 |  |
|  |  |  |  |  | *Model 22.2:* JcDV load + host plant + JcDV load*host plant | 5 | -23.2 | 57.8 | 0.23 |  |
| Survival to adult (Y/N) | 2017 | GLMM | 35 | None | *Model 23.1:* PO activity + melanization + total hemocytes | 5 | -21.6 | 55.2 | 0.06 |  |
|  |  |  |  |  | *Model 23.2:* PO activity + melanization + total hemocytes + host plant | 6 | -20.4 | 55.8 | 0.04 |  |
|  |  |  |  |  | *Model 23.3:* PO activity + melanization + total hemocytes + weight | 6 | -17.6 | 50.2 | 0.70 |  |
|  |  |  |  |  | *Model 23.4:* PO activity + melanization + total hemocytes + host plant + weight | 7 | -17.3 | 52.8 | 0.19 |  |

**Table S7.** Effects of host plant species on immune responses of wild-collected *Euphydryas phaeton* caterpillars, including phenoloxidase (PO) activity, melanization, and concentrations of total hemocytes, granulocytes, oenocytoids, and plasmatocytes. The effects of host plant species (*Chelone glabra* or *Plantago lanceolata*), sampling year (2016 or 2017), and larval body weight at the time of immune assessment were evaluated using linear mixed-effects models including random intercepts for sites. In addition, the interaction between host plant species and year was included in the AICc- best models for PO activity and oenocytoids, and the effects of JcDV infection (Y/N) and its interaction with host plant were included in the AICc- best model for plasmatocytes. For PO activity and plasmatocytes, alternative model structures that excluded these additional fixed effects are also reported, as they received a similarly high level of AICc support to the best-fit model. These predictors were not retained in the models for melanization, total hemocytes, or granulocytes, as their inclusion did not improve model fit (Table S3).

|  | **PO Activity** (AICc-best) | | | | |  | **PO Activity** (ΔAICc = 1.81) | | | |  | **Melanization** | | | | | | |  | **Total Hemocytes** | | | |
| --- | --- | --- | --- | --- | --- | --- | --- | --- | --- | --- | --- | --- | --- | --- | --- | --- | --- | --- | --- | --- | --- | --- | --- |
| *Predictor* | *Estimate ± SE* | | *t* | *df* | *P* |  | *Estimate ± SE* | *t* | *df* | *P* |  | *Estimate ± SE* | | *t* | | *df* | | *P* |  | *Estimate ± SE* | *t* | *df* | *P* |
| Host plant | -8.47 *±* 3.61 | | -2.34 | 439 | **0.020** |  | -4.05 *±* 3.34 | -1.21 | 440 | 0.226 |  | -194.17 *±* 86.42 | | -2.25 | | 377 | | **0.025** |  | -3.26 *±* 5.54 | -0.59 | 432 | 0.557 |
| Year | -10.43 *±* 4.08 | | -2.56 | 439 | **0.011** |  | -3.72 *±* 3.00 | -1.24 | 440 | 0.215 |  | -833.90 *±* 62.83 | | -13.27 | | 377 | | **<0.001** |  | 2.84 *±* 5.72 | 0.50 | 432 | 0.620 |
| Body weight | 0.10 *±* 0.03 | | 3.61 | 439 | **<0.001** |  | 0.09 *±* 0.03 | 3.40 | 440 | **<0.001** |  | 1.86 *±* 0.52 | | 3.59 | | 377 | | **<0.001** |  | 0.09 *±* 0.06 | 1.45 | 432 | 0.147 |
| JcDV infection |  | |  |  |  |  |  |  |  |  |  |  | |  | |  | |  |  |  |  |  |  |
| Host plant x year | 13.22 *±* 5.66 | | 2.34 | 439 | **0.020** |  |  |  |  |  |  |  | |  | |  | |  |  |  |  |  |  |
| Host plant x JcDV |  | |  |  |  |  |  |  |  |  |  |  | |  | |  | |  |  |  |  |  |  |
| Marginal *R^2^* | 0.05 | | | | |  | 0.03 | | | |  | 0.33 | | | | | | |  | 0.01 | | | |
| *n* | 454 | | | | |  | 454 | | | |  | 391 | | | | | | |  | 446 | | | |
|  | **Granulocytes** | | | | |  | **Oenocytoids** | | | |  | **Plasmatocytes** (AICc-best) | | | | | | |  | **Plasmatocytes** (ΔAICc = 1.57) | | | |
| *Predictor* | *Estimate ± SE* | | *t* | *df* | *P* |  | *Estimate ± SE* | *t* | *df* | *P* |  | *Estimate ± SE* | *t* | | *df* | | *P* | |  | *Estimate ± SE* | *t* | *df* | *P* |
| Host plant | -2.96 *±* 5.57 | | -0.53 | 292 | 0.596 |  | 5.41 ± 4.15 | 1.31 | 307 | 0.193 |  | -17.08 *±* 7.55 | -2.26 | | 277 | | **0.025** | |  | -10.03 *±* 6.80 | -1.48 | 279 | 0.141 |
| Year | -20.98 *±* 6.47 | | -3.24 | 292 | **0.001** |  | 15.15 ± 4.14 | 3.66 | 307 | **<0.001** |  | 38.24 *±* 7.07 | 5.41 | | 277 | | **<0.001** | |  | 35.79 ± 6.89 | 5.19 | 279 | **<0.001** |
| Body weight | -0.03 *±* 0.08 | | -0.34 | 292 | 0.734 |  | 0.03 ± 0.04 | 0.86 | 307 | 0.393 |  | 0.23 *±* 0.08 | 2.74 | | 277 | | **0.007** | |  | 0.24 ± 0.09 | 2.84 | 279 | **0.005** |
| JcDV infection |  | |  |  |  |  |  |  |  |  |  | -19.60 *±* 9.34 | -2.10 | | 277 | | **0.037** | |  |  |  |  |  |
| Host plant x year |  | |  |  |  |  | -14.15 ± 5.99 | -2.36 | 307 | **0.019** |  |  |  | |  | |  | |  |  |  |  |  |
| Host plant x JcDV |  | |  |  |  |  |  |  |  |  |  | 31.98 *±* 14.17 | 2.26 | | 277 | | **0.025** | |  |  |  |  |  |
| Marginal *R*^2^ | | 0.06 | | | |  | 0.06 | | | |  | 0.22 | | | | | | |  | 0.23 | | | |
| *n* | | 306 | | | |  | 322 | | | |  | 293 | | | | | | |  | 293 | | | |

**Table S8.** Effects of iridoid glycoside sequestration on immune responses of *Euphydryas phaeton* caterpillars. The effects of IG concentration (total concentration of sequestered IGs), IG composition (proportion of aucubin sequestered out of total IGs), host plant species (*Chelone glabra* or *Plantago lanceolata*), and influential two-way interactions on phenoloxidase (PO) activity, melanization, and total hemocyte concentrations were evaluated using linear mixed-effects models including random intercepts for sites. The results of two models are reported for melanization, and the results of three models are reported for total hemocytes, as alternative model structures that varied in their inclusion of two-way interactions terms (host plant species x IG concentration; host plant species x IG composition) were found to receive a similarly high levels of AICc support to the best-fit model for these analyses. These interaction terms were not retained in the model for PO activity, as their inclusion did not improve model fit (Table S5)

|  | **PO Activity** | | | |  | | **Melanization** (AICc-best) | | | | |  | | **Melanization** (ΔAICc = 0.12) | | | | |
| --- | --- | --- | --- | --- | --- | --- | --- | --- | --- | --- | --- | --- | --- | --- | --- | --- | --- | --- |
| *Predictor* | *Estimate ± SE* | *t* | *df* | *P* |  | | *Estimate ± SE* | *t* | *df* | *P* | |  | | *Estimate ± SE* | *t* | *df* | *P* | |
| IG concentration | -3.41 *±* 2.50 | -1.36 | 241 | 0.174 |  | | 67.10 *±* 98.90 | 0.68 | 206 | 0.498 | |  | | -53.07 *±* 58.57 | -0.91 | 207 | 0.366 | |
| IG composition | -26.29 *±* 9.42 | -2.79 | 241 | **0.006** |  | | -59.52 *±* 308.24 | -0.19 | 206 | 0.847 | |  | | -246.68 *±* 283.36 | -0.87 | 207 | 0.385 | |
| Host plant | 1.66 *±* 5.16 | 0.32 | 241 | 0.748 |  | | 875.15 *±* 298.83 | 2.93 | 206 | **0.004** | |  | | 528.21 *±* 193.72 | 2.73 | 207 | **0.007** | |
| Host plant x  IG concentration |  |  |  |  |  | | -183.98 *±* 122.05 | -1.51 | 206 | 0.133 | |  | |  |  |  |  | |
| Host plant x  IG composition |  |  |  |  |  | | -1202.92 *±* 457.71 | -2.63 | 206 | **0.009** | |  | | -898.38 *±* 412.90 | -2.18 | 207 | **0.031** | |
| Marginal *R*^2^ | 0.06 |  |  |  |  | | 0.06 |  |  |  | |  | | 0.06 |  |  |  | |
| *n* | 252 | | | |  | | 219 | | | | |  | | 219 | | | | |
|  | **Total Hemocytes** (AICc-best) | | | |  | | **Total Hemocytes** (ΔAICc = 0.16) | | | | |  | | **Total Hemocytes** (ΔAICc = 0.34) | | | | |
| *Predictor* | *Estimate ± SE* | *t* | *df* | *P* |  | | *Estimate ± SE* | *t* | *df* | *P* | |  | | *Estimate ± SE* | *t* | *df* | *P* | |
| IG concentration | -12.95 *±* 5.44 | -2.38 | 238 | **0.018** |  | | -21.56 *±* 7.98 | -2.70 | 238 | **0.007** | |  | | -12.80 *±* 5.46 | -2.35 | 239 | **0.020** | |
| IG composition | 46.71 *±* 25.02 | 1.87 | 238 | 0.063 |  | | 19.86 *±* 20.20 | 0.98 | 238 | 0.326 | |  | | 23.91 *±* 20.07 | 1.19 | 239 | 0.235 | |
| Host plant | 10.79 *±* 16.29 | 0.66 | 238 | 0.508 |  | | -28.23 *±* 16.87 | -1.67 | 238 | 0.096 | |  | | -8.42 *±* 10.53 | -0.80 | 239 | 0.425 | |
| Host plant x  IG concentration |  |  |  |  |  | | 15.12 *±* 10.08 | 1.50 | 238 | 0.135 | |  | |  |  |  |  | |
| Host plant x  IG composition | -56.16 *±* 37.34 | -1.50 | 238 | 0.134 |  | |  |  |  |  | |  | |  |  |  |  | |
| Marginal *R*^2^ | 0.06 | | | |  | 0.05 | | | | |  | | 0.05 | | | | |  |
| *n* | 250 | | | |  | 250 | | | | |  | | 250 | | | | |  |

**Table S9.** Effects of iridoid glycoside sequestration on differentiated hemocytes of *Euphydryas phaeton* caterpillars. The effects of IG concentration (total concentration of sequestered IGs) and host plant (*Chelone glabra* or *Plantago lanceolata*) on concentrations of granulocytes, oenocytoids, and plasmatocytes were evaluated using linear mixed-effects models including random intercepts for sites.

|  | **Granulocytes** | | | |  | | **Oenocytoids** | | | |  | **Plasmatocytes** | | | | |
| --- | --- | --- | --- | --- | --- | --- | --- | --- | --- | --- | --- | --- | --- | --- | --- | --- |
| *Predictor* | *Estimate ± SE* | *t* | *df* | *P* |  | | *Estimate ± SE* | *t* | *df* | *P* |  | *Estimate ± SE* | *t* | *df* | *P* | |
| IG concentration | -18.37 *±* 6.22 | -2.95 | 138 | **0.004** |  | | -2.32 *±* 3.10 | -0.75 | 155 | 0.454 |  | -14.60 *±* 5.84 | -2.50 | 146 | **0.014** | |
| Host plant | 9.96 *±* 8.24 | 1.21 | 138 | 0.229 |  | | 5.15 *±* 4.83 | 1.07 | 155 | 0.288 |  | -15.33 *±* 9.49 | -1.61 | 146 | 0.109 | |
| Marginal *R*^2^ | 0.06 | | | | |  | 0.01 | | | |  | 0.08 | | | | |
| *n* | 148 | | | | |  | 165 | | | |  | 156 | | | |  |

**Table S10.** Effects of larval immune responses on survival of *Euphydryas phaeton* individuals infected by Junonia coenia densovirus. A binomial generalized linear mixed-effects model was used to evaluate the effects of standing phenoloxidase (PO) activity, implant melanization score, and total hemocyte concentration, along with the covariate of larval body weight at the time of immune assessment, on survival of infected individuals to the adult stage (Y/N). The strength of measured immune responses did not significantly impact the probability of surviving infection.

|  | **Survivorship** | | | |
| --- | --- | --- | --- | --- |
| *Predictor* | *Odds Ratio* | *95% CI* | *z* | *P* |
| PO activity | 0.974 | 0.000-1.007 | -1.48 | 0.139 |
| Melanization | 0.999 | 0.998-1.001 | -0.94 | 0.347 |
| Total hemocytes | 0.988 | 0.970-1.003 | -1.45 | 0.147 |
| Body weight | 1.039 | 1.011-1.079 | 2.36 | **0.018** |
| Marginal *R*^2^ | 0.39 | | | |
| *n* | 35 | | | |
